# A Liquid Biopsy in Bladder Cancer—The Current Landscape in Urinary Biomarkers

**DOI:** 10.3390/ijms23158597

**Published:** 2022-08-02

**Authors:** Milena Matuszczak, Adam Kiljańczyk, Maciej Salagierski

**Affiliations:** Department of Urology, Collegium Medicum, University of Zielona Góra, 65-046 Zielona Góra, Poland; matuszczakmilena@gmail.com (M.M.); asd.kil@interia.pl (A.K.)

**Keywords:** precision oncology, personalized therapy, cancer biomarkers, liquid biopsy, biological fluids, cell-free DNA, cfDNA, exosomes, extracellular vesicles, bladder cancer, tumour markers, prognosis, diagnosis

## Abstract

The non-muscle invasive bladder cancer tends to recur and progress. Therefore, it requires frequent follow-ups, generating costs and making it one of the most expensive neoplasms. Considering the expensive and invasive character of the current gold-standard diagnostic procedure, white-light cystoscopy, efforts to find an alternative method are ongoing. Although the last decade has seen significant advancements in urinary biomarker tests (UBTs) for bladder cancer, international guidelines have not recommended them. Currently, the paramount urgency is to find and validate the test with the best specificity and sensitivity, which would allow for the optimizing of diagnosis, prognosis, and a treatment plan. This review aims to summarise the up-to-date state of knowledge relating to UBTs and new developments in the detection, prognosis, and surveillance of bladder cancer and their potential applications in clinical practice.

## 1. Introduction: Bladder Cancer Issues and Urinary Biomarkers

Bladder cancer (BCa) is the fourth cause of cancer in men and the most common neoplasm of the urinary tract, with estimated new cases of 81,180 and 17,100 deaths for both sexes in 2022 in the United States [1]. 

BCa is sometimes referred to as “the cancer of the environment and age” since it has been documented to be strongly related to toxins, tobacco, and age—with its peak in the eighth decade of life [2,3].

**Figure 1:**
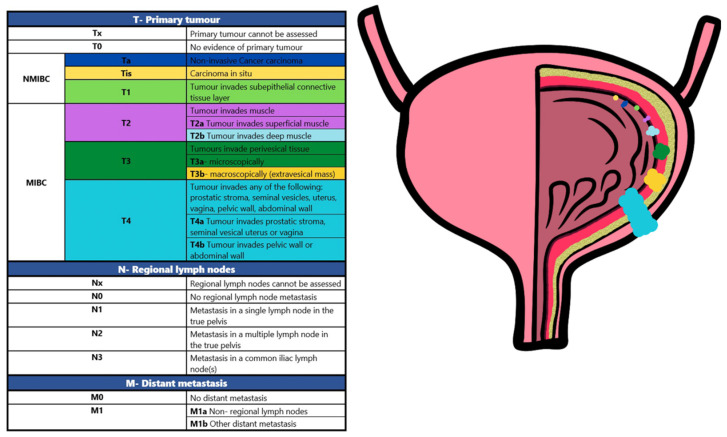
Bladder cancer staging. When the tumour invades the detrusor muscle or beyond (T2-T4 stage), then it is called the muscle-invasive type (MIBC) [4].

BCa at stages Ta/T1/Tis is classified as non-muscle-invasive bladder cancer (NMIBC) (Figure 1) and is a tumour confined to the epithelium of the urinary tract and the underlying lamina propria.

BCa is associated with generally good—long life expectancy, overall low mortality, but also high recurrence rates (up to 80%) and potential progression (approximately 44%) in the clinical course [4,5]. A therapeutic challenge for NIMBC is the frequent recurrence after primary transurethral bladder resection (TURB). The management of NMIBC after TURB consists of intravesical adjunctive treatment and long-term follow-up, which is expensive [6]. Muscle invasion, on the other hand, leads to a dramatic survival prognosis. Patients in this stage typically will only experience a 5-year survival rate of 27–50% [7]. Therefore, regular, and effective monitoring appears to be crucial in managing the disease.

The gold standard for diagnosis and continuous monitoring of BCa remains invasive cystoscopy [7], often associated with risks of infection, pain, and hematuria. Moreover, White light cystoscopy (WLC) is insensitive to flat lesions [8]. The procedure itself is uncomfortable for patients, contributing to non-compliance with scheduled control visits.

Cytology provides a non-invasive alternative to WLC but is limited by its low sensitivity of detection, especially for low-grade (LG) tumours (only 17%) [9] and dependency on the accuracy of analysis by pathologists [10]. Specificity for high-grade (HG) tumours is reasonable, but cytology is limited by urinary tract infections, kidney stones and intravesical therapy [11]. 

Therefore, the European Association of Urology (EAU) guidelines state that cytology cannot be used to ‘reduce the number of cystoscopies’ [4]. Both procedures have significant limitations and are relatively expensive [12], demonstrating the need for developments in diagnosing BCa.

Furthermore, a study [13] by members of the Society of Urologic Oncologists (SUO) found that cystoscopy and cytology are overused among low-risk patients. 

A publication [14] analysed a cohort of 13,054 patients with LG Ta NMIBC diagnosed between 1 January 2004–31 December 2014 and to find factors related to treatment costs. The results suggest that cystoscopy was used too frequently. Since 2005, international guidelines have recommended de-escalation of follow-up cystoscopy, with advice for no more than three cystoscopies two years after diagnosis. Despite the guideline recommendations, follow-up was too often and was associated with an increase in the annual cost of care over time. These data demonstrate the need to work on a method that provides a comfortable reassurance of disease limitation for patients and physicians, preventing overuse of treatment and surveillance and thus reducing the cost of care.

The perfect method should be a non-invasive, rapid, and painless test [15]. Liquid biopsy, for example, detecting biomarkers in urine, is a promising new approach that can overcome these limitations and be incorporated into current clinical practice.

These features make this method easily repeatable, which supports the early detection of BCa. It is important to remember that early diagnosis allows for faster intervention and therefore reduces recurrence and delays disease progression. Not only diagnostic but also prognostic biomarkers are urgently needed. However, the markers published in the literature do not meet the expectations of replacing cystoscopy due to their low specificity and too high a number of false positives, which may be caused by common hematuria.

The advantage of urine over other body fluids is the complete non-invasiveness of obtaining samples and, in the case of BCa, the fact that the physiological function of the bladder is to store urine. Tumours are in direct contact with the urine, which therefore contains a lot of compounds that naturally seep into it [16]. In addition, biomarkers in urine are not regulated by homeostasis and longer storage time promotes hydrolysis by endogenous proteases. Therefore, urine proteome is relatively simple with little complexity (as most of the circulating protein is filtered through the renal tubules), facilitating testing. 

Despite the apparent advantages and approval by the Food and Drug Administration (FDA) and European Medicines Agency (EMA) [17] of some BCa biomarkers (such as nuclear matrix protein 22 NMP22 (Matritech), bladder tumour antigen BTA STAT & TRAK tests (Polymedco; Cortlandt Manor, NY), UroVysion (Abbott Molecular), (fluorescence in situ hybridisations) ImmunoCyt/uCyt+ and cytokeratins (CK-18, CK-20, and CYFRA 21-1) none have yet been included in international recommendations, and their use in general practice remains rare [16]. In the last two years, new BCa biomarkers such as Adxbladder (Arquer Diagnostics; Sunderland, UK), Bladder EpiCheck (Nucleix; Rehovot, Israel), Cxbladder Monitor, and Triage and Detect (Pacific Edge; Dunedin, New Zealand), Uromonitor (U-Monitor; Porto, Portugal), Xpert bladder cancer (Cepheid; Sunnyvale, CA, USA) have become commercially available [18]. However, their diagnostic value still does not provide satisfactory results to allow their implementation in daily practice. The reason may be the lack of reimbursement and the already mentioned low specificity. This review has tried to bring together novelties concerning urinary bladder biomarkers.

## 2. Methods

A literature review was performed by searching PubMed/MEDLINE database from April 2020 to April 2022 to identify studies on new diagnostic, prognostic, and monitoring biomarkers for BCa (Appendix 1). The search terms included bladder cancer; liquid biopsy; urine; tumour markers; prognosis; diagnosis using search terms database = specific—medical subject headings terms in various combinations appropriate to the research objective.

Papers presenting data in the form of reviews, letters to the editor, editorials, research protocols, case reports, brief correspondence and articles not published in English were excluded. Co-workers checked the literature of all included papers for additional studies of interest. On this basis, articles published before April 2020 were also included (23 articles).

Papers in which scientists did not examine urine were excluded from the review. Publications based on tissue, blood, cell lines and animals were excluded. Articles concerning more than one cancer, e.g., prostate, bladder, and kidney cancer, were omitted. In addition, papers focusing on technical feasibility and specifications of measurement methods rather than bladder cancer and clinical utility were excluded. Publications based on small cohorts, i.e., including fewer than five patients, were also excluded.

Researchers independently extracted the following information from the included articles: author name, year of publication, number of patients, stage and/or grade of cancer, and recurrence rate, as well as sensitivity (Se.), specificity (Sp.), negative predictive value (NPV), and positive predictive value (PPV) to assess predictive ability. All data extraction discrepancies were resolved by consensus with the co-authors.

## 3. What Is New in the Last Two Years?

Nowadays, interesting new urinary markers have arrived, based on genetic abnormalities or epigenetic alterations that are commonly found in BCa, such as abnormal DNA methylation, exosomes, metabolites, mRNAs or cell-free DNA (cfDNA). Several of these tests have been tested in the follow-up of patients and have shown very high NPV for NMIBC recurrence.

### 3.1. Methylation

DNA hypermethylation in the promoter regions of tumour suppressor genes can lead to their inactivation and contribute to cancer development. Hypermethylation of the DNA is considered one of the earliest events in urinary tract epithelial carcinogenesis. Therefore, its analysis is a promising tool for the prompt detection of BCa.

The detection and preoperative stratification of BCa based on a urine DNA methylation test was investigated in a publication [19]. Scientists validated the test of two markers (Table 1) and a stratification model in patients with hematuria and suspected BCa. Results of 88.1% and 91.2% sensitivity, 89.7% and 85.7% specificity, respectively, were obtained for these two cohorts. This test also outperformed cytology and fluorescent in situ hybridization (FISH) with respect to sensitivity in detecting LG tumour malignancy (66.7–77.8% vs. 0.0–22.2%, 0.0–22.2%), Ta tumour (83.3% vs. 22.2–41.2%, 44.4–52.9%) and NMIBC (80.0–89.7% vs. 51.5–52.0%, 59.4–72.0%) in both cohorts. Also, in terms of diagnosis of cases with genitourinary co-morbidities, it proved more accurate (88.9–95.8%) compared to cytology (55.6–70.8%) and FISH (72.2–77.8%). In a cohort with suspected BCa, the test with a five-methylation marker detection model (Table 1) identified high-risk NMIBC and muscle invasive bladder cancer (MIBC) with 90.5% sensitivity and 86.8% specificity. 

The authors concluded that DNA methylation-based tests are a highly sensitive and specific approach to address the clinical need for non-invasive diagnosis and prognosis of BCa.

The aim of the study [20] was to validate the diagnostic performance results of 9 methylation markers (Table 1) in urine sediment and full micturition urine. In addition, the diagnostic capacity of the best-performing marker panel from the exploratory study (GHSR/MAL) was evaluated in the preclinical setting. Urinary levels of all methylation markers were significantly higher in BCa patients than in controls (all, *p* < 0.001). Randomised analysis on all nine markers resulted in diagnostic performance with a sensitivity of 81% and specificity of 93%. The same research group previously published an exploratory study relying on methylation of the same nine biomarkers (Table 1), where their area under the curve (AUC) were compared to confirm the technical efficiency of DNA methylation analysis in urine sediment. Similar Receiver operating characteristic (ROC) were obtained in both studies, but the AUC in the current publication was superior. An optimal panel of GHSR/MAL markers was established in the previously mentioned exploratory study. This marker panel reached an AUC of 0.89, corresponding to a sensitivity of 80% and a specificity of 93% (Table 1). The values received were similar; therefore, GHSR/MAL was established as the optimal marker panel to discriminate between BCa patients and controls. The sensitivity of the GHSR/MAL marker panel was determined in different subgroups of BCa patients. These subgroup analyses showed that sensitivity increased with higher tumour grade (G3 vs. G1-G2, HG vs. LG), higher tumour stage (≥T2 vs. Ta/T1/Tis) in primary vs. recurrent tumours and men vs. women. The sensitivity of the GHSR/MAL marker panel was 59% in women and 89% in men (*p* = 0.001). The diagnostic performance of the GHSR/MAL marker panel was also better in primary tumours than in recurrent tumours. Bladder tumours have a higher mass at primary diagnosis than at recurrence, resulting in a superior sensitivity of the GHSR/MAL marker panel in primary tumours. The present findings support further investigation into the clinical value of methylation testing in the primary detection of BCa and the follow-up of patients with NMIBC to reduce the number of invasive cystoscopies.

The Bladder EpiCheck test (Nucleix Ltd., San Diego, CA, USA) (BE) developed from 15 urinary genomic biomarkers (Table 1) based on DNA methylation changes has been [21] tested to replace urine cytology during early follow-up of NMIBC. Patients with HG NMIBC treated with intravesical Bacillus Calmette-Guerin (BCG) and mitomycin C therapy and assessed at follow-up by urine micturition cytology and WLC were included in the prospective study. After three months of follow-up, BE (85.1%) achieved similar specificity to cytology (86.3%). Patients with papillary HG NMIBC had higher specificity in the test group (96.3%) than for cytology (90.4%). Both groups had the same specificity for carcinoma in situ (CIS) (81.4%). The sensitivity of BE was always superior to cytology (for both CIS and HG NMIBC). These results show that this test is superior and can successfully replace cytology.

The aim of the study [22] was to investigate the diagnostic potential of a panel of five hypermethylated gene promoters (Table 1) in urine samples and subsequent determination by quantitative Methylation Specific PCR (qMSP). All genes had a previously described association with BCa development.

The values obtained are similar to the specificity and sensitivity of the Ras Association Domain Family Member 1 (RASSF1) promoter gene alone (86.4% and 52.3%, respectively). The sensitivity of the panel (61.4%) is lower versus cystoscopy for all types of BCa (68–83%) but higher compared to cytology, especially for LG tumours (50%). Moreover, the specificity (86.4%) is significantly higher than cytology for patients with LG cancer and comparable to the specificity of invasive cystoscopy. The panel’s PPV and NPV predictive values were estimated to be 90% and 53%, respectively, and AUC = 0.76. A diagnostic panel was considered positive if it contained at least one methylated promoter. The only promoter gene that was methylated in the control group was RASSF1. Its methylation alone may be a single candidate biomarker for predicting BCa patients compared to controls. The cost-effectiveness of hypermethylation of specific genes for BCa diagnosis in urine samples is favourable. This study suggests that methylation of the proposed panel of genes may be a promising urinary biomarker for BCa diagnosis, but this needs to be confirmed in validation studies in different human populations to develop a ‘universal’ or ‘generic’ test that can detect essentially any BCa. It would be intriguing to test in the future whether this test could correlate with pharmacological responses to drugs conventionally used to treat BCa.

Based on the published literature, researchers selected potentially high-throughput methylation-based panels to detect BCa in urine. In general, panels with more markers were more sensitive but less specific for BCa. Nevertheless, the combination of Protocadherin-17 (PCDH17) and (POU 4 transcription factor 2) POU4F2 showed high sensitivity and specificity and attracted the interest of researchers. Based on this [23], a new panel of three biomarkers (PCDH17, POU4F2 and PENK (Proenkephalin)) was constructed. This panel in detecting BCa achieved 100% specificity and 71% sensitivity, and 100% specificity and 84% sensitivity (Table 1) when combined with the qMSP platform.

### 3.2. Exosomes

Exosomes are small (30–100 nm) membrane vesicles secreted by different types of cells that have a close genetic relationship with endothelial cells. 

They transport various substances: proteins, lipids, and nucleic acids (e.g., mRNA, miR) present in the “parental” cell. However, due to the selectivity of the molecules packed into them, their composition is not identical to that of the cytoplasm. Among other things, membrane adhesion and transport proteins, cytoskeleton components, lysosomal markers, antibody presentation factors, membrane receptors, cytokines, heat shock proteins, and numerous enzymes have been found in exosomes. In addition to the “universal” set of membrane and cytosolic proteins, proteins related explicitly to cellular functions are observed within the proteome of exosomes. The presence in exosomes of specific proteins released by cancer cells indicates the existence of a mechanism for targeted sorting of these molecules, and individual proteins can be regarded as markers indicating the origin of the exosomes and the functional state of the cell that released them. A positive correlation has been shown between the number of exosomes and the stage of disease, supporting their importance in tumour progression. Tumour development is a complex process in which the immune system plays a role as early as the appearance of the first tumour cells. Unfortunately, in the case of tumours, numerous mechanisms have been developed that result in reduced efficiency of the immune system due to, among others, rapid growth of the tumour, the disappearance of the presented antigens on the cancer cell or frequent changes of the presented antigen. One of the functions attributed to exosomes is precisely the inhibition of the immune response in response to a developing tumour, e.g., by initiating the conversion of immune cells into immune suppressor cells. Exosomes may facilitate tumour escape from immune surveillance and promote tumour growth by transferring signalling molecules between cells modulating the tumour microenvironment. Both exosomes released by normal cells and tumour cells are found in the urine of patients with the diagnosis. 

The unique molecular profile of exosomes derived from urine and produced by BCa cells may serve as a “liquid biopsy”. Knowing the components of exosomes would allow the study of the function of these vesicles in the development of this cancer.

In the study [24], the source of tumour differentially expressed genes (DEGs) and the functionality of exosomal RNAs in BCa diagnosis was investigated. The data showed that several urinary exosome DEGs originated from immune cells and displayed a common immune function at the pathway level, reflecting that urinary exosomes were intensively involved in immune activities during cancer initiation and progression.

Finally, 19 genes (Table 2) were selected that had excellent values of AUC = 0.899 for BCa, indicating the good predictive ability of the model. Having a tumour-promoting function, CD248 and MT-ATP were elevated in BCa. A trend in DEAD-Box Helicase 17 (DDX17) expression was also observed (i.e., DDX17 expression in MIBC > DDX17 expression in NMIBCa > DDX17 expression in control samples). This phenomenon may be attributed to the fact that the human RNA helicase DDX17 contributes to tumour cell invasiveness (mainly by regulating alternative folding of DNA and chromatin binding factors).

Another publication [25] focused on the diagnostic properties of exosomes. Their findings highlighted the potential of urinary exosomal mRNA and lncRNA profiling for early diagnosis and provided new insights into the molecular mechanisms involved in BCa. RNA sequencing revealed eight mRNAs and 32 lncRNAs that were elevated in the deeply infiltrating region of the tumour. Five significantly upregulated RNAs (Table 2) were selected for further investigation in urinary exosomes after validation with The Cancer Genome Atlas (TCGA) data. The combination of these RNAs correlated with tumour stage and hematuria severity and had the highest AUC = 0.924 for BCa detection.

Another research group [26] verified two diagnostic models based on exosomes: CDC5L, ITIH2, AFM, CFL1, APOA1, A2M, FGB and CD5L proteins were selected as biomarkers (Table 2). In previous studies, most of these have shown differential expression in BCa, but their clinical validation is still ongoing. 

The area under the two models’ receiver operating characteristic curve (AUROC) outperformed the urinary cytology and was respectively: 0.845 and 0.842. This indicates that these models are more accurate than cytology and highlights the possibility of diagnosing BCa.

A publication [27] investigated the expression of exosomal miRNA-96-5p and miRNA-183-5p (Table 2) in urine as potential biomarkers of BCa. ROC analysis showed that each miRNA had good sensitivity and specificity in differentiating BCa from patients without BCa miR-96-5p 80.4% and 91.8% and miR-183-5p 78.4% and 81.6%, respectively, compared with cytology (37.3% and 100%). Furthermore, their combined sensitivity and specificity were elevated and reached 88.2% and a specificity of 87.8%, respectively. It was shown that miR-96-5p and miR-183-5p could be helpful to non-invasive diagnostic markers in combination with each other and/or urine cytology. Moreover, overexpression of miR-183 correlated strongly with high malignancy and pathological stage of BCa. This may have an additional advantage in clinical practice- in evaluating whether to give adjuvant intravesical chemotherapy to low-risk patients or adjuvant BCG immunotherapy to higher-risk patients. This shows promise for developing an effective therapeutic strategy (based on miRNAs) for the treatment of BCa. 

Another article identified [28] elevated levels of miR-93-5p and miR-516a-5p (Table 2) in the urine of BCa patients compared to healthy controls, which Real-time (RT) qPCR verified. The detection efficiency of BCa miR-93-5p and miR-516a-5p was further evaluated with the AUC= 0.838 and 0.790, respectively, which was significantly better than that of urine cytology (AUC = 0.630). Thus, miR-93-5p and miR-516a-5p and their combinations were shown to be promising biomarkers for diagnosing BCa. In the BCa cohort, miR-93-5p levels were significantly higher in patients with MIBC than NMIBC, and the ROC suggested that miR-93-5p has a promising AUC = 0.769 for distinguishing between MIBC and NMIBC. It was also shown that BCa patients’ miR-93-5p expression levels in urinary exosomes were significantly associated with TNM staging (Ta-T1 vs. T2-T4). 

Bioinformatics analysis revealed that the B cell translocation gene 2 (BTG2) might be a major target gene of miR-93-5p (associated with BCa patient prognosis). MiR-93-5p may act as a tumour promoter in BCa-promotes proliferation, migration and invasion and a novel down-regulatory compound for BTG2 gene expression. In vitro experiments suggested that overexpression of miR-93-5p may contribute to BCa progression by suppressing BTG2 expression. This study found that miR-516a-5p was overexpressed in tissues and urinary exosomes of BCa patients. Urinary-derived miR-516a-5p has the potential to be a non-invasive biomarker for BCa. It can distinguish between MIBC and NMIBC, thereby reducing dependence on bladder examination using a telescopic camera inserted through the urethra. 

Proteomic analysis of extracellular vesicles in urine (EVs) is an up-and-coming method for discovering potential biomarkers of BCa. However, urine contains numerous EVs derived not only from the bladder but also from the kidney and normal urothelial epithelium (which may falsify the results). Researchers in a study [29] attempted to isolate tissue exudate EVs (Te-EVs) from freshly excised live BCa tissue from a culture medium and analyse them proteomic along with EVs from urine. In this way, more reliable BCa biomarkers with high specificity and sensitivity were obtained. This is proven by the fact that Te-EVs contain minimal contaminants such as whole-body proteins in contrast to body fluid samples. After combined proteomic analysis, candidate proteins were verified. 

Multiple reaction monitoring (MRM)/Selective Reaction Monitoring (SRM) analysis showed that levels of six proteins (Table 2) were significantly elevated in urine EVs from BCa patients compared to healthy subjects. The AUC of these six proteins for the diagnosis of BCa ranged from 0.706 to 0.813. HSP90 had the highest AUC value (0.813) of the six proteins, with a sensitivity of 82.5% and specificity of 70.0%. The second highest AUC value (0.785) was obtained for SDC1, with a sensitivity of 82.5% and specificity of 63.3%, followed by MARCKS (AUC = 0.772) with a sensitivity of 65.0% and specificity of 80.0%. All these urinary EV proteins showed better diagnostic capacity than urine cytology (with 48.7% sensitivity and 100% specificity). SRM/MRM analysis showed better AUC and higher levels for HSP90, SDC1 and MARCKS for HG than LG BCa diagnosis. However, there was no association between overall survival (OS) and expression levels of these proteins in BCa patients from TCGA cohort. Although promising, such a method is expensive (SRM/MRM analysis) and time-consuming (ultracentrifugation), limiting clinical application. Furthermore, the cohort of patients without BCa did not include patients with hematuria and cystitis. As these are common symptoms in clinical practice, validation of these biomarkers in such a population is expected.

Promising results were achieved in a publication [30]; however, this was conducted on a very small population. Therefore, these reports should be verified by a larger group in the future. Urinary exosomes have been investigated for the clinical relevance of urinary levels of the telomerase component of lncRNA (TERC). TERC carries the potential to play a role as a diagnostic and prognostic biomarker of BCa. Indeed, the results indicated a significant predominance of TERC in urinary exosomes of BCa patients compared to healthy controls. Urinary exosomal TERC showed higher sensitivity (78.65%) and specificity (77.78%) than existing indices, including nuclear matrix-22 and urine cytometry. The AUC was 0.836 with a cut-off value of 4.302 for exosomal TERC. Furthermore, this non-invasive assay distinguished between LG and HG tumours.

### 3.3. Proteomics

A proteome is the entire set of proteins produced or modified by an organism. Initially, the term was defined as the total protein complement encoded by a given genome. However, nowadays, it also includes any isoforms, post-translational modifications, interactions, and anything that is “post-genomic”. Since proteins manage and/or reflect cellular processes, the study of proteomics provides an attractive pathway for research because it enables the rapid identification of protein profiles in a biological sample. Proteomics generally refers to the large-scale experimental analysis of proteins and proteomes and, in recent times, has enabled the identification of an increasing number of proteins. This depends on the time and different conditions or stresses a cell or organism is exposed to. Proteomics is an interdisciplinary field that uses genetic information from various genomics projects, including the Human Genome Project. Recently, proteomics has expanded into clinical research with goals ranging from elucidating disease pathogenesis to discovering clinical biomarkers. The study of proteomics includes large-scale protein detection, identification, and characterization. The potential applications of this emerging field are vast. They will likely provide significant insights into improving our future understanding and management of cancer, including that addressed in this BCa review.

BCa can progress with epithelial inflammation. APOA1 is a well-known negative marker of inflammation-its levels decrease by over 25% when there is inflammation. Smoking leads to cystitis, resulting in lower urinary APOA1 protein levels and falsifying results because BCa is more common in smokers. Given the potential role of APOA1 in various malignancies, it is speculated that a combined genetic and proteomic analysis of APOA1 may help observe predisposition and aid in the clinical diagnostic stratification of BCa. To this end, a study [31] analysed the association of APOA1-75 G/A and +83 C/T genotypes with BCa predisposition. In addition, we examined APOA1 protein expression in urine samples to find a potential link between differential protein expression in urine and changes in APOA1 genotypes.

The study provided evidence for an important role of APOA-1 in carcinogenesis at BCa’s genetic and protein levels. A strong association was also observed between APOA1-75G/A and bladder tumour risk and its association with protein expression in urine, validating its possible role as a marker for disease risk assessment and a promising diagnostic marker for different grades of BCa malignancy. This is supported by functional studies showing that APOA1 plays a vital role in tumour growth, angiogenesis, invasion, and metastasis.

The research group found BCa with the APOA1-75AA genotype variant more frequently (70.0%) and with higher expression (≥20 ng/mL) in urine and differed significantly compared with wild-type APOA1 GG protein (*p* = 0.03). Lower urinary APOA-1 protein levels showed a significant association with HG tumours (<20 ng; 84.6% versus 15.4%), while LG tumours were associated with six times higher expression (≥20 ng). Among the different BCa stages, APOA-1 showed lower expression (<20 ng/mL) at higher tumour stages, Ta (47.6%), T1 (52.3%) and T2 (70%), respectively. Similarly, higher expression (≥20 ng) showed a decreasing trend with the progression of invasion from Ta, T1 and T2 (52.4%, <47.7% and <30.0%, respectively). The results demonstrate an association between APOA-1 expression and its protective effect on BCa.

In a study [31], proteomic paired pre-and post-operative urine samples from BCa patients were analysed using isobaric tracers for relative and absolute assessment (iTRAQ) based quantitative technology. Comparison of urine samples from the same patients before and after surgery led to the discovery of proteins with significantly differential expression, some of which may prove crucial in the prognosis of progression and become valuable biomarkers for monitoring and predicting BCa. Proteins (Table 3) have been found to correlate with the occurrence and progression of BCa. Changes in their expression may also help assess recurrence.

Publication [32] describes an inexpensive, non-invasive assay to identify fucosylated integrin alpha-3 glycoisoform (ITGA3) directly from untreated urine using lectins coated with europium-doped nanoparticles (Eu 3+-NPs). Evaluation of individual samples showed that the glycovariant ITGA3-UEA assay could significantly distinguish BCa from benign prostatic hyperplasia (BPH) patients (*p* = 0.007). The Ulex europaeus (UEA) agglutinin-I lectin showed increased binding to BCa-derived ITGA3.

Diagnostic and prognostic analysis showed elevated levels of BTA, NMP22, and survivin (Table 3) [33]. The triple combination of survivin + BTA + Cytology was the most promising model to distinguish BCa (AUC = 0.97, sensitivity 67%, specificity 96%). Univariate survival analysis showed that cytology (HR = 5.35) and survivin (HR = 3.24) were significantly associated with progression-free survival (PFS). In addition, survivin (HR = 4.15) was statistically significantly associated with cancer-specific survival (CSS). All biomarkers showed good diagnostic performance, higher sensitivity than cytology but weaker specificity. However, multivariate analysis did not show these markers as independent prognostic factors.

An ELISA-based method for detecting the trans-membrane protein programmed death-ligand 1 (PD-L1) was tested in urine samples from BCa patients [34]. This protein plays a significant role in suppressing the anti-cancer cellular response and is secreted by BCa cells. This study confirmed increased urinary PD-L1 expression in newly diagnosed patients with NMIBC and MIBC (before TURB treatment), and recurrent BCa after treatment (TURB) compared to control subjects. AUC = 0.78 with sensitivity and specificity = 0.65, and 0.95 was highest when detecting newly diagnosed BCa patients. Also, this cohort reached PPV = 0.87 and NPV = 0.84, respectively. Researchers found increased PD-L1 levels in locally advanced BCa.

Furthermore, NMIBC patients with PD-L1 expression are predicted to have an unfavourable response to BCG. It was supposed that PD-L1 could be a reliable biomarker to monitor and help diagnose patients with BCa. Still, it has not yet reached the desired accuracy, so it may be an addition to a multiparametric panel to monitor and diagnose BCa.

In March 2022, the ability of ADXBLADDER to predict the likelihood of BCa recurrence and reduce the frequency of follow-up cystoscopy in LG pTa NMIBC was evaluated [35]. ADXBLADDER is a novel biomarker test that measures MCM5 protein level in urine, and on this basis, we can safely avoid invasive procedures like WLC. This prospective, double-blind, multicentre study enrolled patients undergoing follow-up cystoscopy after TURB/biopsy of suspicious lesions and tested with ADXBLADDER. The accuracy and decision curve analysis of ADXBLADDER and the effect on the decision to perform cystoscopy were examined. The primary endpoint was the NPV in detecting HG recurrence +/− CIS (HG/CIS) and the effect on reducing unnecessary cystoscopies. Results showed that ADXBLADDER had a sensitivity of 66.7% and NPV of 99.15% in detecting HG/CIS recurrence (Table 3). Patients with LG pTa cancer at previous diagnosis (whose ADXBLADDER NPV = 99.15% excludes HG/CIS recurrence) are suitable for a less invasive surveillance strategy with ADXBLADDER and safe omission of cystoscopy. The HG/CIS recurrence probability was 5.0% for patients with a positive and 0.85% for patients with a negative ADXBLADDER result.

Intravesical BCG provides a non-specific immune system stimulator and is an effective therapy for patients with NMIBC. The Oncuria™ is an assay that allows to diagnose, predict and monitor BCa which was investigated in a study [36] to predict response to this live attenuated tuberculosis vaccine. The test was evaluated before treatment with BCG in a cohort consisting of intermediate- to high-risk NMIBC and then assessed the test’s ability to identify those patients in whom BCG is ineffective against tumour recurrence. After BCG treatment, patients who developed BCa recurrence had elevated (Oncuria™ panel) levels of ANG, APOE, A1AT, CA9, MMP9, MMP10, PAI1, SDC1, VEGFA (AUC = 0.89, and sensitivity 81.8% and specificity 84.9%). Furthermore, risk factor analysis showed that higher urinary levels of ANG, CA9 and MMP10 were associated with a significantly higher risk of relapse. This demonstrates the potential use of Oncuria™ to assess the utility of intravesical BCG treatment for patients with BCa.

A pilot survey study [37] using Oncuria™ investigated how the test affects physicians’ use of non-invasive and invasive diagnostic tests for microhaematuria, gross haematuria, and BCa surveillance. The results showed that with a negative test result, the total number of diagnostic procedures was reduced by 31% and a positive result by 27%. The test influenced at least one change in management decisions in more than 90% of urologists. In the 10% with a strongly positive test result, urologists were more likely to skip cystoscopy and go directly to a definitive diagnostic procedure such as TURB to expedite patient care, thereby speeding up assessment, treatment and reducing costs.

**Table 3:** Table presenting data for proteomics biomarkers.

Biomarker	Purpose	Number of Patients	Method	Diagnostic Value	Prognostic Value	Predictive Capacity	Type of Study	Reference
APOA1	prognostic	*n* = 258 (BCa = 108; control group = 150)	Genomic DNA was extracted from the blood and tumour tissues of all patients using the phenol chloroform method and DNA extraction kit (Zymo Research Corporation, Irvine, CA, USA). PCR was conducted using DNTP (Sigma-Aldrich, St. Louis, MO, USA), 10 mMdTTP primers (Sigma-Aldrich, St. Louis, MO, USA) and Taq DNA polymerase (Biotools, Madrid, Spain). PCR-RFLP was performed using a restriction endonuclease enzyme (New England Biolabs, NEB, England). APOA1 concentrations were measured using standard source ELISA kit (Thermo Scientific, Waltham, MA, USA).	n/a	BCa progression	AUC = 0.889	retrospective	[38]
CDHR2	Prognostic and surveillance	*n* = 40 (*n* = 8 no history or evidence of BCa, *n* = 4 geneal urinary system diseases, *n* = 16 BCa prior to surgery, *n* = 12 BCa after surgical removal of the tumour)	Urine samples of 40 patients obtained pre- and postoperatively. iTRAQ reagent kit (8plex, Applied Biosystems, Bedford, MA, USA) was used to quantify proteins in the urine. Q-Exactive mass spectrometry (Thermo Fisher Scientific, Waltham, MA, USA) was used to analyse the samples which then were validated with western blot.	n/a	Postoperative non-recurrence probability	No data	retrospective	[31]
HSP27				n/a	High expression is associated with BCa progression	No data		
HNRNPA2B1				n/a	Postoperative non-recurrence probability	No data		
BTA	Diagnostic and prognostic	*n* = 157 (BCa = 61, NMIBC: LG = 30, HG = 15; MIBC: hG = 16; UTUC = 44; control group = 52)	Cytology urine of 157 patients was performed and interpreted by pathologists. NMP and BTA-stat were measured by NMP22 (Alere INC., Waltham, MA, USA) and BTA-stat assays (Polymedco CDP, LLC., NY, USA). BTA was measured by chemiluminescence (Pergrande, Beijing, China), and ELISA for survivin (R&D systems, MN, USA). Data analysis performed with SPSS v.19.0 (IBM Corp., Armonk, NY, USA) and GraphPad prism7 (GraphPad Software Inc., Sand Diego, CA, USA).	BCa vs. UTUC differentiation	BCa survival	AUC = 0.84, PPV = 82%, NPV = 72%, Se. = 74%, Sp. = 81%	retrospective	[33]
BTA-stat						AUC = 0.67, PPV = 70%, NPV = 64%, Se. = 69%, Sp. = 63%		
NMP22						AUC = 0.69, PPV = 77%, NPV = 62%, Se. = 59%, Sp. = 79%		
Survivin						AUC = 0.84, PPV = 84%, NPV = 75%, Se. = 74%, Sp. = 81%		
PD-L1	diagnostic	*n* = 122 (group 1 = 20, NMIBC: LG = 7, HG = 9; MIBC: HG = 4; group 2 = 63, NMIBC: LG = 10, HG = 21; MIBC: LG = 3, HG = 29; group 3 = 39, NMIBC: LG = 17, HG = 30; MIBC: LG = 3, HG = 33)	Quantikine ELISA for Human/Cynomolgus Monkey PD-L1/B7-H1 Immunoassay from R&D Systems (Catalog Number DB7H10) was used to inspect the urine samples of 122 patients. Data analysis was performed with SAS software v 9.4 (SAS institute Inc., Cary, NC, USA).	BCa detection		AUC = 0.74, PPV = 92%, NPV = 47%, Se. = 53%, Sp. = 90%	retrospective	[34]
ADXBLADDER	prognostic	*n* = 629 (No recurrent BCa = 550, recurrent BCa = 79)	ADXBLADDER test was performed on urine samples of 629 patients. Data analysis was performed with Stata 12.1 (StataCorp,.College Station, TX, USA)		BCa recurrence prediction	AUC = 0.56, PPV = 17.5%, NPV = 99.15%, Se. = 66.7%, Sp. = 76.0%	prospective	[35]
ANG	prognostic	*n* = 64	Urine samples of 64 patients were tested with multiplex bead-based immunoassay (Oncuria™) from R&D Systems Inc (Minneapolis, MN, USA). Data analysis was performed with SAS software version 9.3 (SAS Institute Inc., Cary, NC, USA).		Treatment response, recurrence	AUC = 0.7444, PPV = 41.2%, NPV = 91.5%, Se. = 63.6%, Sp. = 81.1%	prospective	[36]
CA9						AUC = 0.6878, PPV = 30.4%, NPV = 90.2%, Se. = 63.8%, Sp. = 69.8%		
MMP10						AUC = 0.7238, PPV = 32%, NPV = 92.3%, Se. = 72.7%, Sp. = 67.9%		

Abbreviations: AUC—Area under the ROC Curve, *n*—number of patients participating in study, HG—high grade, LG—low grade, BCa—bladder cancer, PCR—polymerase chain reaction, PCR-RFLP—Restriction fragment length polymorphism PCR, PPV—positive predictive value, NPV—negative predictive value, Se.—sensitivity, Sp.—specificity, APOA1—Apolipoprotein A1, CDHR2—Cadherin Related Family Member 2, HSP27—heat shock protein beta-1, HNRNPA2B1—Heterogeneous Nuclear Ribonucleoprotein A2/B1, BTA—Bladder Tumour Antigen, NMP22—Nuclear Matrix Protein-22, PD-L1—Programmed Cell Death 1 Ligand 1, ANG—Angiogenin, CA9—Carbonic Anhydrase 9, MMP10—Matrix Metallopeptidase 10.

### 3.4. mRNA

Messenger RNA (mRNA) is a single-stranded RNA involved in protein synthesis and is formed from a DNA template during the transcription process. The role of mRNA is to carry protein information from the DNA in the cell nucleus to the cell’s cytoplasm, where the mRNA sequence is read, and protein synthesis occurs. mRNA, like any other RNA, can undergo changes that affect the proteins produced. Cancer may be associated with, among other things, decreased levels of proteins that destroy tumour cells or increased levels of those that promote cancer cell division. This discovery has further emphasised the importance of abnormal protein synthesis in oncogenesis. Temsirolimus modulates signalling pathways regulating this process (it exerts an inhibitory effect on mTOR (mammalian target of rapamycin) with a regulatory function in the cell cycle. It leads to inhibition of translation of essential regulatory proteins of the G1 phase of the cell cycle preceding replication) and is an effective anti-cancer drug.

A study [39] indicates that mRNA expression of ROBO1, CRH and IGF2 (Table 4) correlates with an increased risk of BCa. Medium-risk cancers were detected in 81.8% and high risk in 100%. The decision curve analysis showed that the 3-marker panel is more effective than the currently used cystoscopy in diagnosing BCa, especially in the screening population (95.0% of intermediate- and high-risk BCa were detected, and 91.2% among patients undergoing follow-up). Analysis of these three urinary biomarkers in detecting intermediate- and high-risk BCa (these stages included together as “increased-risk” disease) showed a specificity of 73.5% and a sensitivity of 92.5%. A reliable measure of clinical utility is undoubtedly the number of cystoscopies avoided. The use of the panel avoided 149 cystoscopies, missing only one patient with intermediate-risk disease. The panel could therefore prove helpful in selecting patients who can safely avoid the examination and those who will benefit from imaging technologies. Clarifying individual cystoscopy appointments will reduce the burden of this examination on patients and the health care system and focus more on higher-risk patients who may benefit from early and intensive treatment. 

Analysis of BCa cell lines [40] of the RNA transcriptome revealed linear and circular transcripts of calcium-binding protein S100 6 (S100A6) and translocation-related membrane protein 1 (TRAM1) as highly promising potential markers for this disease. This study showed that the S100A6/TRAM1 ratio has a high ability to diagnose BCa based on urinary RNA. In addition, the authors point out that RNA is an early, potentially useful diagnostic marker and speculate that studying circular forms of transcripts specific for S100A6 and TRAM1 may be even more promising. In addition, they note the added advantage of focusing on tumour biology and the potential for developing gene-targeted drug therapy.

The Xpert bladder cancer Monitor (Xpert BC Monitor) (Cepheid, Sunnyvale, CA, USA) is a qualitative in vitro diagnostic test developed to monitor BCa recurrence. The Xpert BC Monitor assay measures levels of five target mRNAs (Table 4) by performing a real-time reverse transcriptase-polymerase chain reaction (RT-PCR). The study [41] was designed to evaluate the performance of the Xpert BC Monitor in routine clinical practice during follow-up of patients with NMIBC. All patients with previously diagnosed NMIBC underwent standard care during the study period were eligible. Patients who underwent TURB or BCG treatment within six weeks before study inclusion were excluded.

For Xpert BC Monitor, the overall sensitivity and specificity were 72.7% and 73.7%, respectively. For cytology Se. and Sp. were 70.7% and 97.8%, respectively. The overall NPVs were 96.5% for Xpert BC Monitor and 92.8% for cytology. After excluding LG recurrences, the sensitivity of Xpert BC Monitor increased to 92.3%, with NPV reaching 99.7% (330/331). ROC curves showed that the diagnostic performance of Xpert BC Monitor (AUC = 0.73) was higher than that of cytology (AUC = 0.53), especially for the detection of HG tumours (AUC = 0.83 vs. AUC = 0.55). The advantage of this prospective study was the lack of patient selection, which more reliably reflects clinical reality.

A prospective study [42] that investigated the role of the urine mRNA based Xpert BC test in predicting tumour recurrence. For this purpose, patients with NIMBC after TURBT were studied to predict a positive repeat biopsy for malignancy. A urine sample was collected two to six weeks after primary TURBT for Xpert BC analysis, and patients were eligible for repeat biopsy. Patients with benign pathology, incomplete resection, coexisting CIS/urinary tract epithelial tumour or MIBC were excluded. A positive Xpert BC test after primary total NMIBC resection is significantly associated with a positive repeat biopsy for malignancy (66.4% of positive results showed a positive repeat biopsy (HR = 6.2)).

Furthermore, the Xpert BC test is an independent predictor of early cancer recurrence. In multivariate Cox regression analysis, the Xpert BC test was significantly associated with cancer recurrence (HR = 9.7). The sensitivity, specificity, PPV and NPV of the Xpert BC test for repeat biopsy were: 85.9%, 72.3%, 66.4% and 88.9%. During the follow-up period with a median of 12 months, tumour recurrence occurred in 35% of patients. 

The Xpert BC test can be used after the initial resection of NMIBC to minimise unnecessary repeat biopsies with potential healthcare cost savings and reduced patient morbidity.

To preoperatively assess whether cancer spreads into the detrusor muscle of the bladder [43] analysed gene expression using microarrays between NMIBC and MIBC. The study highlighted the role of CYR61 in cell migration and invasion. Higher CYR61 expression in MIBC was found in tumour samples using qRT-PCR and ELISA (on average 2.5-fold compared with NMIBC). The sensitivity for distinguishing MIBC from NMIBC was 72.7% and specificity 86.0%; therefore, CYR61 can be considered a promising biomarker for the preoperative diagnosis of MIBC.

A study [44] evaluated the performance of a novel narrow-band imaging (NBI)-based cystoscopy technology and a new urine test, the Xpert BC Monitor, for early detection of recurrent NMIBC. Then were compared with standard diagnostic controls. 

The results obtained were for cytology: se = 27%, sp = 97%, PPV = 65% and NPV = 87%. For cytology combined with WLC: se = 28%, sp = 98%, PPV = 86% and NPV = 79%. For Xpert BC Monitor se = 58%, sp = 89%, PPV = 51%, NPV = 92% and AUC = 0.79. 

Subgroup analysis showed enhanced HG scores in the Xpert BC Monitor group. LG se and sp were 33% and 74%, while HG se and sp were 89% and 89% (LG PPV and NPV were 21% and 94%, respectively, while HG PPV and NPV were 39% and 97%, respectively). NBI cystoscopy does not contribute to improved outcomes compared to standard WLC. However, as mentioned above, the Xpert BC Monitor may provide improved sensitivity and diagnostic advantage in cases of HG recurrence, suggesting its utility in the early detection of potentially harmful recurrences. Given the technological ability to develop such a platform over time, the Xpert BC Monitor remains a method that may be useful in the future.

A longitudinal prospective cohort study [45] showed that for patients under active surveillance (AS) for recurrent bladder cancer, the Xpert BC Monitor avoids cystoscopy and urine cytology. The study was designed to enrol patients under AS protocol for recurrent NMIBC as participants in the Bladder Cancer Italian Active Surveillance (BIAS) project. Their urine samples were analysed using the Xpert BC Monitor at the time of inclusion in the study. Patients who were Xpert BC Monitor negative underwent additional Xpert BC Monitor testing at 4, 8 and 12 months. The clinical utility of the Xpert BC Monitor study was assessed by determining the number of cystoscopies and cytologies that could be avoided within one year. The results showed that two consecutive negative test results avoided 73.9% unnecessary biopsies (with a 26.4% risk of failure). In contrast, four negative tests were associated with preventing 100% of biopsies (with only a 12% risk of failure). All patients with a negative test had negative urine cytology. At a median follow-up of 23 months, failure-free survival (FFS) stratified by 0, 1 or ≥2 negative tests were: 67.0, 55.1 and 84.1. 

A pilot study [46] compared CxBladder (Cxb) (Table 4) achieved by reverse RT-qPCR urine cytology as an adjunct to surveillance cystoscopy (CS) after resection of NMIBC.

During CS (dependent on operator skill with risk of missed lesions), supplementation with urine cytology is often used but lacks sensitivity and specificity in detecting recurrence (in this study, cytology results were only 13% se and NPV 74%, while sp and PPV was 100% in predicting a positive cystoscopy result). The study compared common practice with adding a new mRNA test (Cxb) to cystoscopy. Cxb showed a sensitivity and NPV of 100%, a specificity of 75% and a PPV of 62% in predicting a positive cystoscopy result. Due to its high NPV and sensitivity, CxBladder predicts suspicious cystoscopy findings very well and has great potential in NMIBC surveillance when used as an adjunct to cystoscopy.

The diagnostic efficacy of the Cxbladder Resolve (CxbR) test, alone and in combination with other tests (Cxb Triage (CxbT) and Detect (CxbD)), was evaluated [47] in a population of patients with hematuria. The results allowed us to assign patients to a high-risk group of high-impact tumours (HIT; HG Ta, Tis or T1-T3). In the HIT group, CxbR had a sensitivity of 92.4% and a specificity of 93.8% (Table 4). External validation of the Cxb tests showed that NPV = 99.4%, excluding 87.6% of patients from further testing. With a specificity of 96.3%, all (100%) HIT were correctly identified, with three LG tumours missed. In both studies reported in this publication, all HIT patients were correctly assigned in priority.

### 3.5. Mutations

However, literature [48] suggests that some biomarkers cannot be helpful (Table 5). In a prospective study of bladder cancer, mutations of the PIK3CA and AKT1 genes, the main players in the PI3K-AKT-mTOR signalling pathway (which plays a significant role in cell growth, proliferation, and survival), were examined. The results obtained and their statistical analysis did not show a significant association; therefore, these biomarkers were considered of low value because too rare events trigger them. Researchers from Morocco demonstrated that they did not correlate with clinical and pathological data in the study population.

In a pilot study [49] TERT promoter mutations in urine were detected by Droplet Digital PCR. Diagnostic accuracy was evaluated in patients with ongoing oncology and after resection at risk of BCa recurrence. AUC, sensitivity, and specificity were: 0.768, 55.56 and 100%, respectively. Sixty patients were included in the study, of whom 27 patients had histologically confirmed BCa; 23 patients had no symptoms of BCa (control group); 10 patients underwent TURB 3–6 months before urination (the “second look” group). In positive samples, the fraction of tumour DNA varied significantly from 0.59 to 48.77%. In the “second look” group, tumour DNA was detected in 4/10 patients, indicating the possibility of BCa recurrence, whose fraction varied from 0.90 to 6.61%. Therefore, liquid biopsy based on urinary TERT promoter mutations seems to be a promising tool for diagnosing and surveillance BCa.

In a study published in April this year [50], researchers, through targeted ultra-deep urine DNA sequencing, have sought to detect gene mutations associated with BCa in a highly sensitive and specific manner. Urine DNA mutation analysis diagnosed BCa with 87.3% sensitivity and 84.8% specificity in two independent cohorts with hematuria. The sensitivity was 97.4% for grade 3, 86.5% for grade 2 and 70.8% for pTa BCa grade 1. Among patients under NMIBC surveillance, tumour recurrence was detected with a sensitivity of 86.2% with a specificity of 62.5%. A positive urine mutation test alone (in the absence of clinically detectable disease) was associated with a 2.6-fold increased risk of future recurrence. Although a limitation of the study was the low number of recurrences in the NMIBC control group and the low sensitivity for pTa BCa grade 1, this panel is a promising tool for patients with hematuria for BCa detection and NMIBC surveillance.

Genome-wide association studies (GWAS) have identified genetic variants associated with BCa predisposition in European and Chinese populations. In a study [50], two variants, rs9642880 and rs710521, were evaluated in urine to determine the association between the rs9642880 and rs710521 genotypes and the risk of BCa development and progression in a previously unstudied Egyptian population. Results showed an association of the TT genotype rs9642880 G>T and AG genotype rs710521 A>G with BCa risk.

**Table 5:** Table presenting data for mutations biomarkers.

Biomarker	Purpose	Number of Patients	Method	Diagnostic Value	Prognostic Value	Predictive Capacity	Type of Study	Reference
PIK3CA	diagnostic	*n* = 70 (LG = 27, HG = 43)	Genomic DNA extracted from fresh frozen tumours and urine cell sediments of 70 patients using phenol/chloroform method. DNA was quantified with a NanoDrop 2000 Spectrophotometer (Thermo Fisher Scientific, Waltham, MA, USA). DNA amplification was performed on ProFlex PCR system (Applied Biosystems, Foster City, CA, USA). Sequencing was performed with BigDye Terminator v3.1 Cycle Sequencing Kit (Applied Biosystems, Foster City, CA, USA). Data analysis IBM SPSS software version 23.	BCa detection		Se. = 66.7%, Sp. = 100%	prospective	[48]
AKT1						Se. = 100%, Sp. = 100%		
TERT	diagnostic	*n* = 60 (BCa (NMIBC) = 27, LG = 16, HG = 6; control group = 23; *n* = 10, tmr)	DNA was isolated from urine samples of 60 patients using QIAamp Circulating Nucleic Acid Kit (Qiagen GmbH, Hilden, Germany). The reference plasmids were created using pUC19 vector (cloning sites KpnI and HindIII). Forward primer and reverse primer were provided by Evrogen RU, AO. DNA isolated from liver cancer cells (originating from the HepG2 cell line; cat no. 85011430; MilliporeSigma) was used for amplification of the mutant C228T fragment. High-Fidelity DNA Polymerase (New England BioLabs Inc., Ipswich, MA, USA) was used to amplify the mutant insert. Quantification of DNA was performed using the QX200 ddPCR System (Bio-Rad Laboratories, Inc., Hercules, CA, USA). Detection of TERT promoter and mutations was achieved using TaqMan Liquid Biopsy dPCR Assays (TERT_C228T, Assay ID Hs000000092_rm and TERT_C250T, Assay ID Hs000000093_rm; Thermo Fisher Scientific Inc.). Data analysis performed with IBM SPSS Statistics 22.0 Software (IBM Corp., Armonk, NY, USA).	BCa detection		AUC = 0.768, Se. = 55.56%, Sp. = 100%	retrospective	[49]
DNA sequencing	diagnostic	Retrospective haematuria clinic cohort = 214 (BCa = 97, non-BCa = 117)	DNA extraction was performed with Quick-DNA urine kits (D3061; Zymo Research, Irvine, CA, USA) on urine samples collected from all patients and quantified using high-sensitivity dsDNA Qubit kits (Thermo Fisher, Waltham, MA, USA). Libraries were prepare using Nonacus Cell3 Target enrichment and sequenced on a NovaSeq (Illumina, San Diego, CA, USA).	BCa detection		Se. = 87.6%, Sp. = 88.9%	retrospective	[50]
		Prospective haematuria clinic cohort = 215 (BCa = 68, non-BCa = 147)				Se. = 86.8%, Sp. = 81.0%	prospective	
		NMIBC surveillance cohort = 293 (BCa = 29, non-BCa = 264)				Se. = 86.2%, Sp. = 62.5%	prospective	
		Control group = 162 (normal samples = 100, confirmatory control samples = 62)				Normal samples Sp. = 89.9% Confirmatory control samples Sp. = 91.2%	retrospective	
GWAS (rs9642880, rs710521)	prognostic	*n* = 200 (BCa = 150, NMIBC = 12, MIBC = 123; control group = 50)	DNA was extracted from urine of 200 patients with Qiagen Kits (Hilden, Germany) and then measured on a Nanodrop ND-2000c (Thermo Scientific, Waltham, MA, USA). PCR was carried out using Bio-RAD T100Thermal cycler. Thermo Scientific FastDigestStyl was used to digest. Data analysis performed with Microsoft Excel 2016 and IBM SPSS Statistics for Windows, version 26 (IBM Corp., Armonk, NY, USA).		BCa progression	No data	retrospective	[51]

Abbreviations: AUC—Area under the ROC Curve, *n*—number of patients participating in study, HG—high grade, LG—low grade, BCa—bladder cancer, PCR—polymerase chain reaction, PPV—positive predictive value, NPV—negative predictive value, Se.—sensitivity, Sp.—specificity, PIK3CA—Phosphatidylinositol-4,5-Bisphosphate 3-Kinase Catalytic Subunit Alpha, AKT1—AKT Serine/Threonine Kinase 1, TERT—Telomerase Reverse Transcriptase, GWAS—Genome-wide association studies.

### 3.6. Metabolites

By analysing urinary metabolites, researchers [52] identified potential biomarkers to help differentiate hematuria from BCa (AUC: 0.781) (Table 6). Furthermore, eight markers different BCa at an early stage with an AUC of 0.976. They also contained information about BCa samples with hematuria and those without hematuria. Of these eight metabolites, four were: D-ribose, D-fructose, D-mannose, and D-galactose are pentoses and hexoses rarely found in the urine of healthy people. Their high content in the urine of BCa patients may suggest a correlation between sugar metabolism and BCa carcinogenesis. Two others are sugar alcohols-ribitol and erythritol. So, most of these putative markers seem closely related to the interaction of sugar metabolism and polyols. Although high blood sugars may not directly suggest diabetes, some studies have shown a link between diabetes and BCa risk. 

Ultra-performance liquid chromatography coupled to mass spectrometry (UPLC-MS) was used [53] to profile urinary metabolites of BCa patients. Eleven potential biomarkers were extracted with a combined AUC = 0.983, and sensitivity = 95.3% and specificity = 100% (Table 6). These results showed excellent discriminatory ability for distinguishing BCa from healthy controls.

### 3.7. Others

The researchers verified the hypothesis that cells excreted in the urine of BCa patients could also be optically imaged by targeting VPAC1 receptors covalently bound to a fluorophore-labelled peptide. Their study [54] showed encouraging results for VPAC receptor positivity-its sensitivity (89.23%) was superior to conventional cytology (63.07%) and fluorescent cytology (87.69%). The specificity was 100%, which matched conventional cytology (100%) and beat 5-ALA-induced fluorescent cytology (90.47%). The data they obtained are very encouraging and warrant further research to develop a simple and reliable tool for the non-invasive detection of BCa.

The standard treatment for MIBC is neoadjuvant chemotherapy followed by radical cystectomy, which significantly affects the quality of life. The inability to non-invasively assess minimal residual disease (MRD) limits the ability to offer bladder-sparing treatment. In the study [55], researchers tried to find a non-invasive method to assess MRD and improve bladder-sparing treatment. Urine tumour DNA (utDNA) was evaluated. For this purpose, they used personalized urine cancer profiling by deep sequencing (uCAPP-Seq) in cell-free DNA (cfDNA) urine samples obtained on the day of radical cystectomy with curative intent from 42 patients with localized BCa. The results showed that the median utDNA level was 0% in healthy adults and 2.4% in BCa patients. Detection of utDNA MRD was strongly correlated with a lack of pathologic complete response (pCR) (*p* < 0.001), with a sensitivity of 81% and specificity of 81%. UtDNA MRD-positive patients showed significantly worse PFS than utDNA MRD-negative patients (HR = 7.4). Patients with high TMB (tumour mutational burden) may take advantage of early treatment (with checkpoint blockade-personalized immunotherapy for BCa).

Researchers [56] found that urinary ceruloplasmin (CP) levels were associated with tumour grade and pT stage in patients with NMIBC. CP expression in BCa tissues was positively associated with tumour growth and progression. CP levels were significantly higher in the urine of BCa patients compared to healthy subjects and patients with benign diseases such as urinary tract infections and stones. Furthermore, elevated urinary CP levels were independently associated with shorter periods without recurrence. In multivariate analysis, Kaplan-Meier survival curves showed that positive CP expression significantly predictor recurrence and OS. High urinary CP levels were also identified as a significant predictor of recurrence in patients with NMIBC (HR = 2.87). Based on the results, the investigators concluded that CP plays a vital role in tumour growth, progression and survival in BCa.

To evaluate the response to standard NMIBC therapy with intravesical BCG infusion, we performed a utility test as a prognostic marker of relapse and progression after BCG treatment, the Mycobacterium tuberculosis complex PCR (MTC-PCR) in urine [57]. For this purpose, patients were observed for the presence of a positive MTC-PCR result. Most patients were positive at least once during follow-up. The group that was not positive was associated with significantly worse five-year relapse-free survival and disease progression (*p* < 0.001). Using multivariate Cox regression analysis, it was shown that MTC-PCR positivity at least once was a significant predictor of recurrence (HR = 36.782) and progression (HR = 47.209). Therefore, the absence of a positive result may be a valuable biomarker for predicting progression and relapse after BCG treatment.

Angiogenin (ANG) is one of the proteins most involved in tumorigenesis. It supports the Folkman hypothesis that tumour growth depends on angiogenesis. An angiogenic agent is involved in many cellular processes, including development and division. It is also the first human tumour-derived protein to develop blood vessel growth. Tumour formation is a multi-step process involving cells’ genetic and epigenetic changes and selectively supports the neoplasm microenvironment. Available data show that ANG affects almost all stages of tumour formation, including tumour cell survival, proliferation, migration and invasion, and angiogenesis.

For this reason, a meta-analysis [58] of ANG as a potential marker of BCa was performed. It showed a pooled sensitivity of 0.71, specificity of 0.78, and AUC of 0.789 (Table 7), which gives a chance to use ANG in diagnosing BCa. However, these results need further verification in studies on more numerous cohorts.

Researchers [59] investigated the ability of biomarkers to discriminate between patients with malignant and non-malignant bladder disease using 3D HPLC (high-performance liquid chromatography) with an absorbance and fluorescence detector. A set of seven chromatographic peaks (five absorbances plus two fluorescence) with high classification power (100% sensitivity and 100% specificity) were evaluated for discriminating false positives due to haematuria from true BCa. The differentiation model (OPLS-DA) diagnosed BCa with 100% sensitivity and 96% specificity. Monitoring chromatographic peaks with absorption and fluorescence detection demonstrated the potential for a non-invasive diagnostic test (Table 7). In addition, the application of this method is cheap, fast, and effortless and presents high accuracy, according to the publication. The proposed method could therefore be a good outpatient tool for diagnosis, therapeutic progression, and prevention of recurrence of BCa.

A study investigated miRNA profiles and surface-enhanced Raman spectroscopy (SERS) of urine to develop diagnosis and stratification biomarkers [60]. Evaluation using machine learning algorithms was applied to distinguish the BCa group from the healthy group. Subsequently, molecular stratification of LG and HG tumours and between luminal and basal types was performed based on miRNA and SERS profiling. In discriminating between BCa and healthy cohort, three expressed miRNAs (miR-34a-5p, miR-205-3p, miR-210-3p in combination with SERS gave better accuracy (AUC = 0.92) (Table 7) than for each method alone (AUC = 0.84). Also, when assessing classification accuracy, the collaboration of both methods gave a better result (AUC = 0.95) than each method alone (miRNA AUC = 0.89, SERS AUC = 0.92). Based on the above results, it was concluded that SERS profiling synergises with miRNA and achieves better diagnostic capabilities.

Analysis of urinary volatile organic compounds (VOCs) in a cross-sectional study [61] compared the profiles of patients with and without BCa and revealed potential signatures of diagnostic feasibility. Urine samples were collected from clinic patients with hematuria (21 had a new diagnosis of BCa, 125 had no cancer) undergoing diagnostic cystoscopy and patients with BCa undergoing follow-up (75 had recurrent BCa, 84 had no recurrence). A characteristic VOC profile was observed in BCa patients compared with controls. Two predictive models were evaluated using internal validation. A panel of six VOC biomarkers achieved AUC = 0.80 and sensitivity = 0.71 and specificity = 0.80. A panel of 8 VOCs had slightly worse results AUC = 0.77 and sensitivity = 0.71 and specificity = 0.72, respectively (Table 7). Urinary VOC analysis has the potential to influence future clinical practice by assisting in diagnosis and monitoring. Significant trends in urinary VOCs enable the identification of some but not all BCa samples from clinically relevant controls.

## 4. Discussion

Protein biomarkers’ overall sensitivity and specificity are between urine cytology and cystoscopy. Their sensitivity decreases significantly when we use them to detect BCa at an early stage. A possible reason for the decrease in sensitivity is that their concentration in early-stage bladder cancer is usually low. This makes it difficult to detect them in urine samples and reduces the chance of identifying patients with cancer. The main argument against biomarkers is their low specificity, but as the data collected in this publication (Table 1, Table 2, Table 3, Table 4, Table 5, Table 6 and Table 7) show, the specificity is satisfactory and, for some, superior to cystoscopy.

The top of the iceberg is the early identification of intermediate- and high-risk BCa patients with a high tendency to recur and progress. 

All exosomes showed better diagnostic parameters in comparison to cytology. Additionally, their overexpression correlated with the tumour grade and allowed differentiation of the pathological stage of BCa. These properties made it possible to personalise therapy—tailoring treatment to the tumour stage. In addition, it is hoped that exosomes will, in the future, enable the development of a method for non-invasive monitoring of tumour progression/regression in real-time treatment and predicting patient prognosis.

Satisfactory diagnostic values were also obtained for proteomics biomarkers. Attention is drawn to commercially available tests: ADXBLADDER, CxBladder or Oncuria.

It has been shown [36] that patients diagnosed with LG pTa are ideally suited for less intensive surveillance using this test. Patients with LG pTa at a previous diagnosis whose ADXBLADDER NPV = 99.15% excludes HG/CIS recurrence are suitable for a less invasive surveillance strategy using ADXBLADDER and safe omission of cystoscopy. It helps reduce unnecessary cystoscopy procedures in negative patients and thus potentially improves the quality of life and overall healthcare costs.

Due to its high NPV and sensitivity, CxBladder predicts suspicious cystoscopy findings very well and has great potential in NMIBC AS when used as an adjunct to cystoscopy. Furthermore, CxbR [47] has high sensitivity and specificity, correctly identifying all high-impact tumour (HIT; HG Ta, Tis or T1-T3) risk groups. Sequential Cxb tests stratify patients with a low or high probability of HIT with a 4.8-fold higher diagnostic yield than stratification according to the American Urological Association guidelines. Oncuria™ [36,37] has proven to be a test that can rule out the presence of BCa, reduce healthcare costs and improve patient outcomes.

The authors [19,20,21,22,23] concluded that DNA methylation-based assays are a highly sensitive and specific approach that can provide a reliable source of precision medicine and contribute to the genomic diagnosis of BCa.

Differences in age, gender, tumour type, nature: primary or recurrent and different ways of tumour evaluation (only some of them reviewed by pathologists) influence the variety of results obtained.

Worse diagnostic efficacy results were observed in women. A study [20] showed a marked difference between the sensitivity of the GHSR/MAL marker panel in men and women, while its specificity was similar in both sexes. The authors concluded that the methylated DNA in female urine is diluted due to the large amount of unmethylated DNA from normal cells of gynaecological origin [62,63]. Furthermore, the presence of leukocytes may also cause high background values of unmethylated DNA in women’s urine [63,64]. It has been suggested that a possible method to increase the sensitivity of the GHSR/MAL marker panel in women is to use midstream urine, as gynaecological cells are mainly present at the beginning of micturition [65]. Another possible method to overcome gender-related differences is to use different cut-off thresholds for men and women.

The diagnostic potential of metabolites was highlighted in the study [53], a breakthrough that opens a potentially new direction for biomarkers. In addition to genetic or protein markers, metabolites are applicable biomarker candidates due to their role in various pathways. Comparative urinary metabolomics has been used to identify BCa metabolites such as lactate, phosphocholine and adenosine [16].

Identification and quantification are essential steps in metabolomics. Analytical chemists have developed high-performance techniques for the identification and quantification of compounds or molecules in omics studies, such as liquid chromatography-mass spectrometry (LC-MS), gas chromatography-mass spectrometry (GC-MS) or nuclear magnetic resonance (NMR). 

The advantage of chromatography is its speed, low cost of performing and the small amount of work it requires—the results of the publication [16] present high accuracy. Therefore, the proposed method could be an excellent ambulatory tool for diagnosis, therapeutic progression, and prevention of BCa recurrence. Of all, two-dimensional gas chromatography coupled to a time-of-flight mass spectrometer (GC × GC-TOFMS) achieves the best analytical results [66]. A study comparing the metabolic profiles of urine from BCa patients and healthy subjects using GC × GC-TOFMS has been performed. It provides information on several metabolite markers such as taurine, citrate and succinate [67].

Molecular urinary markers may improve the understanding of the evolution of BCa and their micro and macroenvironment, thus contributing to improved prognostic and predictive outcomes. In addition, stratifying patients into specific risk groups enables tailored interventions.

The fact that detection performance improves with the number of markers, and some BCa was only detected by one particular panel, suggested significant inter-individual heterogeneity of BCa. Heterogeneity of disease is a vital element that can lead to divergent results when looking at biomarkers in urine. Heterogeneity of BCa exists on at least several levels: inter-individual (genotypic) variations between patients, inter-tumour differences between primary tumour and metastasis, intra-tumour differences-different regions of the tumour have distinct genetic variations, tumour variations over time +/− during/period of treatment. In addition to tumour genotype, tumour cell metabolism is highly variable. The tumour microenvironment is related to the emission of environmental signals. Differences in nutrient and oxygen levels can cause changes in metabolism. Such limitations in nutrient availability can cause changes in the phenotype of the cell, and the tumour with a nutrient limitation can progress. It has been observed that different stages and types of BCa are associated with different characteristic metabolism. For example, tryptophan metabolism is elevated in the urine of HG compared to LG NMIBC patients.

Despite numerous limitations, which are open to debate, the new papers published bring optimism to the field of BCa diagnosis and prognosis. “Liquid biopsy” of the urine is a method that allows the continuity of follow-up visits (invasive techniques are associated with non-compliance with the schedule) and influences faster diagnosis, which contributes to more immediate treatment and a reduction in the costs associated with the treatment of advanced MIBC. Optimising therapy using these non-invasive tools reduces complications such as infection, pain or haematuria and appears cost-effective. In addition, tests based on urinary biomarkers show the same or even higher sensitivity with primary lesions [20], thus missing the risk of overlooking flat lesions associated with cystoscopy.

The results of the works mentioned above allow better patient stratification, which optimises therapy. In the case of low-risk patients, it will enable decisions to be made as to when to administer adjuvant intravesical chemotherapy and, in the case of higher-risk patients, when to administer adjuvant BCG immunotherapy. In patients with NMIBC and PD-L1 expression, an unfavourable response to BCG is predicted [34].

## 5. Summary

Identifying patients at the highest risk who may benefit most from aggressive early treatment remains crucial for the uroncologist. Cancer causes a systemic response, which causes metabolic and immunological changes that biomarkers can measure.

The future will tell whether the high NPV of UBC will be acceptable to patients and sufficiently valuable for urologists. Patients appreciate the apparent high sensitivity of cystoscopy despite its discomfort. Before a urinary biomarker test is recommended as a control (instead of CS), the diagnostic values should be superior to or close to cystoscopy. Research is also needed to develop a geographically matched diagnostic biomarker for BCa.

## Figures and Tables

**Table 1:** Table presenting data for methylation biomarkers.

Biomarker	Purpose	Number of Patients	Method	Diagnostic Value	Prognostic Value	Predictive Capacity	Type of Study	Reference
VIM, OSTM1, SLC4A10, AC092805.1, ONECUT2	Prognostic	Cohort 1 = 192 (116 BCa, MIBC = 47, NMIBC = 68; 76 non-BCa)	Urine samples collected from 464 patients before cystoscopy or surgery. Genomic DNA was extracted using the QIAamp DNA blood Mini Kit (Qiagen, Germany, Catalog No. 51106) and quantified by the Qubit Assay (Thermo Fisher Scientific, USA, Catalog No. Q32851). Bisulfite treatment was performed on genomic DNA with the EZ-96-DNA Methylation-Direct MagPrep Kit (Zymo Research, USA, Catalog No. D5044). The methylation of bisulfite-treated DNA was analyzed by a 22-marker BCA DNA (AnchorDX, China, Catalog No. UME043) on the QuantStudio 3 Real-Time PCR System (Thermo Fisher, Waltham, Ma, USA). EpiTect PCR Control DNA Set (Qiagen, Germany, Catalog No. 59695) was used as positive and negative controls. DNA quantified by the Meth-Quant Master Mix (AnchorDx, China, Catalog No. UME043-01 ) and the 22-marker BCA Detect Panel (AnchorDx, China, Catalog No. UME043-02).		Preoperative risk stratification of BCa	**Non-BCa** AUC = 0.78, PPV = 88.6%, NPV = 81.8%, Se. = 87.8%, Sp. = 82.9%	Retrospective	[19]
						**LMR-NMIBC** AUC = 0.78, PPV = 54.5%, NPV = 91.7%, Se. = 46.2%, Sp. = 93.9%		
						**NMIBC+MIBC** AUC = 0.78, PPV = 80.4%, NPV = 84.8%, Se. = 83.1%, Sp. = 82.4%		
		Cohort 2 = 98 (59 BCa, MIBC = 22, NMIBC = 35; 39 non-BCa)				**Non-BCa** AUC = 0.821, PPV = 81.1%, NPV = 87.2%, Se. = 91.1% Sp. = 87.2%		
						**LMR-NMIBC** AUC = 0.821, PPV = 54.5%, NPV = 90.5%, Se. = 42.9%, Sp. = 93.8%		
						**NMIBC+MIBC** AUC = 0.821, PPV = 84.4%, NPV = 92.0%, Se. = 90.5%, Sp. = 86.8%		
ONECUT2, VIM	Diagnostic and prognostic	Cohort 1 = 192 (116 BCa, MIBC = 47, NMIBC = 68; 76 non-BCa)		BCa detection		AUC = 0.898, PPV = 88.6%, NPV = 80.8%, Se. = 87.1%, Sp. = 82.9%	Retrospective	
		Cohort 2 = 98 (59 BCA, MIBC = 22, NMIBC = 35; 39 non-BCa)				AUC = 0.921, PPV = 92.9%, NPV = 83.3%, Se. = 88.1%, Sp. = 89.7%		
		Cohort 3 = 174 (35 BCa, MIBC = 5, NMIBC = 29; 147 non-BCa)				AUC = 0.935, PPV = 60.8%, NPV = 97.6%, Se. = 91.2%, Sp. = 85.7%	Prospective	
FAM19A4	diagnostic	*n* = 208 (BCa = 108; LG = 45, HG = 63; Control group = 100, haematuria = 34, benign urological conditions = 43, healthy = 23)	Urine samples of 208 patients were collected before cystoscopy or TURBT. DNA was isolated from urine using QIAamp DNA Mini Kit (Qiagen GmbH, Hilden, Germany). DNA concentrations were measured with NanoDrop 1000 (ThermoFisher Scientific, Waltham, MA, USA). EZ DNA Methylation^™^ Kit (Zymo Research, Orange, CA, USA) was used for bisulphite conversion. qMSP was performed to identify methylation values of targeted biomarkers. Data analysis were performed with R Statistical Software (v.3.6.1, R Foundation for Statistical Computing, Vienna, Austria).	BCa detection		AUC = 0.72	Retrospective	[20]
GHSR						GHSR AUC = 0.89, MAL AUC = 0.85, GHSR/MAL AUC = 0.89, Se. = 80%, Sp. = 93%		
MAL								
miR-129						AUC = 0.83		
miR-935						AUC = 0.79		
PHACTR3						AUC = 0.69		
PRDM14						AUC = 0.88		
SST						AUC = 0.84		
ZIC1						AUC = 0.88		
Altogether						Se. = 81%, Sp. = 95%		
Bladder epicheck	diagnostic	*n* = 205 (HG NMIBC = 135, T1G3 = 86, T1G2 = 49, CIS = 70)	Urine samples collected from 205 patients were centrifuged twice. DNA was extracted using Bladder EpiCheck DNA extraction kit and prepared for the PCR assay using the Bladder EpiCheck test kit. Data analysis performed with GraphPad-Prism 5 software (Graphpad Software-Prism 5 software, San Diego, CA, USA) and MedCalc version 10.2.0.0 (MedCalc Software, Mariakerke, Belgium).	BCa detection		AUC = 0.94, PPV = 60%, NPV = 97.7%, Se. = 94.3%, Sp. = 79.6%	Prospective	[21]
DNA hypermethylation (RASSF1, RARB, DAPK, TERT, APC)	diagnostic	*n* = 85 (BCa = 50, NMIBC = 37, MIBC = 5; control group = 35)	Urine samples were collected form 85 patients before cystoscopy. DNA was extracted from urine using the Cells and Tissue DNA Isolation Kit (Norgen Biotek Corp., Thorold, Canada). Sodium bisulfite modification of DNA was performed with the EZ DNA Methylation-GoldKit (Zymo Research, Orange, CA). Quantification of the percentage of methylation of DNA was performed with Luna Universal Probe qPCR Master Mix (New England Biolabs, Ipswich, MA, USA) and on Applied Biosystems StepOnePlus Real Time PCR System (Thermo Fisher Scientific, Inc., Waltham, MA, USA).	BCa detection		AUC = 0.76, PPV = 90%, NPV = 53%, Se. = 61.4%, Sp. = 86.4%	Prospective	[22]
PCDH17, POU4F2, PENK	diagnostic	*n* = 252 (panel design: BCa = 18, control group = 15; validation: BCa = 107, control group = 100)	DNA from the urinary cell pellets was extracted using QIAamp DNA Mini Kit (Qiagen). DNA quality and quantity were assessed using a NanoDrop2000 (Thermo Scientific, Wilmington, DE, USA). Bisulfite conversion was performed with a ZYMO EZ DNA Methylation-Gold Kit (ZYMO, Irvine, CA, USA). Sequencing was performed on an Illumina Hiseq platform. The bisulfite modification reaction was executed by 96-Well GeneAmp PCR System 9700 (Applied Biosystems, Foster City, CA, USA). DNA amplification was performed on 7500 Real-Time PCR System (Applied Biosystems, Foster City, CA, USA).	BCa detection		AUC = 0.96, Se. = 87%, Sp. = 97%	Retrospective	[23]

Abbreviations: AUC—Area under the ROC Curve, *n*—number of patients participating in study, HG—high grade, LG—low grade, BCa—bladder cancer, PCR—polymerase chain reaction, PPV—positive predictive value, qPCR—quantitative polymerase chain reaction, NPV—negative predictive value, Se.—sensitivity, Sp.—specificity, VIM—Vimentin, OSTM1—Osteoclastogenesis Associated Transmembrane Protein 1, SLC4A10—Solute Carrier Family 4 Member 10, AC092805.1—, ONECUT2—One Cut Homeobox 2, FAM19A4—TAFA chemokine like family member 4, PHACTR3—Phosphatase And Actin Regulator 3, PRDM14—PR/SET Domain 14, SST—Somatostatin, ZIC1—Zic Family Member 1, RARB—Retinoic Acid Receptor Beta, DAPK—Death Associated Protein Kinase 1, TERT—Telomerase Reverse Transcriptase, APC—Adenomatous Polyposis Coli, miR—microRNA.

**Table 2:** Table presenting data for exosomes.

Biomarker	Purpose	Number of Patients	Method	Diagnostic Value	Prognostic Value	Predictive Capacity		Type of Study	Reference
**Exosome 2 (CD248, MT-ATP)**	Diagnostic	*n* = 116	Urine samples were collected from 116 patients and then expression of genes and functionality of exosomal RNAs were investigates by using single-cell mapper (scMappR). Data analysis performed with R package (version 3.0.2, R-project).	BCa detection		AUC = 0.898		Retrospective	[24]
KLHDC7B	Diagnostic and prognostic	*n* = 180 (group 1 = 10 BCa, LG = 4, HG = 6; 10 HCs, group 2 = 80 BCa, LG = 35, HG = 45; 80 HCs)	Urine samples collected from 90 patients. Extraction of exosomes was conducted using a commercial kit (Norgen Biotek Corp., Thorold, Canada). Nanoparticle Tracking Analysis (NTA) was used to examine the size distribution and concentration of exosomes (ZetaView particle tracker, ZetaVIEW S/N 17-310, Particle Metrix, Germany). Extracted exosomes were imaged using a JEM-1400 transmission electron microscope (JEOL Inc., Peabody, MA, USA). Western Blot was performed using Western Chemiluminescent HRP Substrate (WBKLS0100). Urine Exosome RNA Isolation Kit (Norgen Biotek Corp, Product No. 47200, Thorold, Canada) was used to extract total exosome RNA, and then evaluated by a NanoDrop spectrophotometer (Thermo Fisher Scientific, Waltham, MA, USA). RT-qPCR was performed using SYBR Premix Ex-Taq II (RR820A, Takara, Dalian, China). Data analysis performed using SPSS 24.0 software (IBM Corp., Armonk, NY, USA).	BCa detection	BCa progression	AUC = 0.842, PPV = 68.5%, NPV = 86.7%, Se. = 68.5%, Sp. = 88.3%		Retrospective	[25]
CASP14						AUC = 0.765, PPV = 80.3%, NPV = 64.7%, Se. = 77.5%, Sp. = 70.6%			
PRSS1						AUC = 0.823, PPV = 60.3%, NPV = 87.5%, Se. = 78.1%, Sp. = 75.0%			
MIR205HG						AUC = 0.843, PPV = 56%, NPV = 88.7%, Se. = 77.3%, Sp. = 83.1%			
GAS5						AUC = 0.729, PPV = 74.7%, NPV = 64.1%, Se. = 78.7%, Sp. = 60.3%			
A2M	Diagnostic and surveillance	*n* = 156 (discovery = 12; *n* = 24, LG = 3, HG = 3; verificatio*n* = 24, LG = 0, HG = 18; validatio*n* = 120, LG = 20, HG = 75)	Urine protein and exosome was extracted from 156 participants urine. Prepared urinary peptides were analyzed using liquid chromatography-tandem mass spectrometry LC-MS/MS. MS raw files were processed in MaxQuant (v.1.5.3.1). Data-dependent acquisition (DDA) and data-independent acquisition (DIA) methods were conducted with an Ultimate 3000 UHPLC system (Dionex, Sunnyvale, CA, USA) coupled to a Q-Exactive Plus mass spectrometer (Thermo Fisher Scientific Inc., Waltham, MA, USA).	BCa detection	n/a	A2M AUC = 0.658 CFL1 AUC = 0.629 ITIH2 AUC = 0.759 Model 1(3 biomarkers) AUC = 0.845, PPV = 48.5%, NPV = 94.9%, Se. = 88.0%, Sp. = 81.3%	Model 2 (All biomarkers) AUC = 0.842, PPV = 42.5%, NPV = 95.8%, Se. = 85.0%, Sp. = 74.7%	Retrospective	[26]
CFL1									
ITIH2									
APOA1						AUC = 0.702			
AFM						AUC = 0.687			
FGA						AUC = 0.612			
CDC5L						AUC = 0.659			
CD5L						AUC = 0.658			
miRNA-96	Diagnostic	*n* = 100 (study group = 72, NMIBC = 22, MIBC = 29; control group = 28)	Exosomes were isolated from urine of 100 patients before treatment using miRCURY Exosome Isolation Kit (Qiagen, Hilden, Germany). Extraction of total miRNA was performed with miRcute miRNA isolation kits (Tiangen biotech, Beijing, China). miScript Reverse Transcription Kit (Qiagen GmbH, Hilden, Germany) was used for reverse transcription and polyadenylation of the miRNA to complementary DNA (cDNA). Quantification of exosomal miRNA was performed with a Stratagene Mx3005P. Statistical analysis was performed using SPSS software (IBM Corp., Armonk, NY, USA).	BCa detection		AUC = 0.85, PPV = 91.1%, NPV = 81.8%, Se. = 80.4%, Sp. = 91.8%		Prospective	[27]
miRNA-183						AUC = 0.83, PPV = 81.6%, NPV = 78.4%, Se. = 78.4%, Sp. = 81.6%			
miR-93-5p	Diagnostic	*n* = 120 (BCa = 12, NMIBC = 6, MIBC = 6; control group = 4; validatio*n* = 104, BCa = 53, control group = 51)	Exosomes were isolated from urine collected from 120 patients before treatment in an ultracentrifuge (Beckman Coulter, Miami, FL, USA). Exosomes were processed for nanoparticle tracking analysis (NTA) with NanoSight NS300 instrument (Malvern, UK). Total protein was extracted in RIPA lysis buffer (89,900, Thermo Fisher Scientific, Waltham, MA, USA). Total RNA was extracted with the Trizol Reagent (15,596,026, Invitrogen, Carlsbad, CA, USA). RNA was quantified and assessed by NanoDrop ND-2000 (Thermo Fisher Scientific, Waltham, MA, USA). miRNA expression was quantified using TaqMan single^®^ microRNA assays (442,975, Applied Biosystems^®^, Foster City, CA, USA). qRT-PCR was performed with ABI 7300 Real-Time PCR System (Applied Biosystems, Foster City, CA, USA). Data analysis was peformed using R 3.5.1 (R Foundation for Statistical Computing, Vienna, Austria).	BCa detection		AUC = 0.838, Se. = 74.1%, Sp. = 90.2%		retrospective	[28]
miR-516a-5p						AUC = 0.790, Se. = 72.9%, Sp. = 89.9%			
HSP90	diagnostic	*n* = 81 (discovery phase: BCa = 7, NMIBC = 3, MIBC = 4; control group= 4; validation phase: BCa = 40, NMIBC = 20, MIBC = 20, control group = 30)	Urine samples were collected from 77 and BCa tissue samples from 47 patients. Proteis concentration of Te-EVs was measured using a Micro BCa protein assay kit (Thermo Fisher Scientific, Waltham, MA, USA). Proteins in urinary and tissue samples were separated using SDS-PAGE and transferred on to a polyvinylidene difluoride (Thermo Fisher Scientific, Waltham, MA, USA). The size and concentration of EVs were analyzed using NTA system (NanoSight). TEM was used to inspect the samples with JEM-1400Plus transmission electron microscope (JEOL Ltd., Tokyo, Japan). EVs were lysed using a MPEX PTS reagent kit (GL Science). TMT 10-plex system (Thermo Fisher Scientific, Waltham, MA, USA) was used for TMT-labeling. TMT-labeled peptides were alanyzed using a Q-Exactive Plus mass spectrometer (Thermo Fisher Scientific, Waltham, MA, USA) with an UltiMate 3000 Nano-flow high-performance LC system (Dionex, Sunnyvale, CA, USA) and an HTC-PAL autosampler (CTC Analytics, Zwingen, Switzerland). Data analysis was performed using JMP Pro software (v.14.0.0, SAS Institute, CARY, N.C, USA), and visualization quantification was performed using GraphPad Prism software (v.7.05; GraphPad Software, San Diego, CA, USA).	BCa detection		AUC = 0.813, Se. = 82.5%, Sp. = 70.0%		retrospective	[29]
SDC1						AUC = 0.785, Se. = 82.5%, Sp. = 63.3%			
MARCKS						AUC = 0.772, Se. = 65%, Sp. = 80%			
MARCKSL						AUC = 0.757,			
TJP2						AUC = 0.748			
CD55						AUC = 0.706			
TERC	Diagnostic and prognostic	*n* = 128 (LG = 20, HG = 108)	Exosomes used for sequencing were isolated from urine using differential centrifugation. Exosomes used for validation were isolated from urine using exosome extraction kit (BestBio, Shanghai, China). TEM was performed to view and capture images (Thermo Fisher Scientific, Waltham, MA, USA). NanoSight LM10 system (Malvern Instruments LTD., Malvern, UK) was used to detect the concetration and size distribution of particles. Western blot was performed using anti-TSG101 (Abcam, Cambridge, UK), anti-HSP70, antiAnnexin V, and anti-CD9 (Cell Signaling Technology, Danvers, MA, USA). Second antibody antirabbiy IgG (Millipore, Burlington, MA, USA). RNA sequencing was performed using an Illumina Novaseq 6000 system (San Diego, CA, USA). qPCR was performed using TB Green Premix Ex Taq II (Tli RNaseH Plus, Takara, Dalian, Japan) on an Applied Biosystems 7300 real-time PCR system (Waltham, MA, USA). Data analysis perfomed with Graphpad Prism 8 (GraphPad Software Inc., Sand Diego, CA, USA) and MedCalc v.15.2.2 (MedCalc software Ltd., Ostend, Belgium).	BCa detection		AUC = 0.836, Se. = 78.65%, Sp. = 77.78%		retrospective	[30]

Abbreviations: AUC—Area under the ROC Curve, *n*—number of patients participating in study, HG—high grade, LG—low grade, BCa—bladder cancer, PCR—polymerase chain reaction, PPV—positive predictive value, qPCR—quantitative polymerase chain reaction, RT-qPCR—Real-time quantitative polymerase chain reaction NPV—negative predictive value, Se.—sensitivity, Sp.—specificity, CD248—Tumour Endothelial Marker 1, MT-ATP—Mitochondrially Encoded ATP Synthase Membrane Subunit 6, KLHDC7B—Kelch Domain-Containing Protein 7B, CASP14—Caspase 14, PRSS1—Serine Protease 1, MIR205HG—MIR205 Host Gene, GAS5—Growth Arrest Specific 5, A2M—Alpha-2-Macroglobulin, CFL1—Cofilin 1, APOA1—Apolipoprotein A1, ITIH2—Inter-Alpha-Trypsin Inhibitor Heavy Chain 2, AFM—Afamin, FGA—Fibrinogen Alpha Chain, CDC5L—Cell Division Cycle 5 Like, CD5L—CD5 Molecule Like, miR—microRNA, HSP90—Heat Shock Protein 90, SDC1—Syndecan 1, MARCKS—Myristoylated Alanine Rich Protein Kinase C Substrate, MARCKSL—MARCKS-related Protein, TJP2—Tight Junction Protein 2, CD55—Complement Decay-accelerating Factor, TERC—Telomerase RNA Component.

**Table 4:** Table presenting data for mRNA biomarkers.

Biomarker	Purpose	Number of Patients	Method	Diagnostic Value	Prognostic Value	Predictive capacity	Type of Study	Reference
ROBO1, CRH, IGF2	diagnostic	*n* = 177 (screening = 95; surveillance = 76; both = 6)	Urine collected from 177 patients and then evaluated d for ROBO1, WNT5A, CDC42BPB, ABL1, CRH, IGF2, ANXA10, and UPK1B expression using GeneXpert Dx (Cepheid, Sunnyvale, CA, USA) automated multiplex RT-PCR platform. Statistical analysis was performed using statistical software R 3.5 (R Foundation for Statistical Computing).	BCa screening and surveillance (detection)	Risk stratification	AUC = 0.923, PPV = 47.1%, NPV = 97.4%, Se. = 92.5%, Sp. = 73.5%	retrospective	[39]
S100A6, TRAM1	diagnostic	*n* = 113 (HR patients = 66; control group = 47)	RNeasy Midi Kit (QIAGEN, Hilden, Germany) was used to isolate RNA from urine samples of 113 patients which then were quantified using a NanoDrop ND-1000 spectrophotometer (Thermo Fisher Scientific, Waltham, MA, USA). Measuring of concentration and integrity of pooled urinary RNA was done with Agilent 2100 Bioanalyzer and Agilent RNA 6000 Pico Kit (Agilent Technologies, Santa Clara, CA, USA). cDNA synthesis was done with SMARTer Stranded Total RNA-Seq Kit—Pico Input Mammalian (TaKaRa Bio INC., Kusatsu, prefecture Shiga, Japan) and then purified with Agencourt AMPure XP PCR purification system (Beckman Coulter, Brea, CA, USA). The double-stranded cDNA libraries were quantified with Qubit dsDNA HS Assay Kit and the Qubit Fluorometer (Thermo Fisher Scientific, Waltham, MA, USA). DNA was sequenced by GATC Biotech AG (GATC Biotech AG, Konstanz, Germany). qPCR was performed using SYBR green and TaqMan Systems. Data analysis was performed with SDS 2.1 software (Applied Biosystems, Foster City, CA, USA).	BCa detection		No data	retrospective	[40]
Xpert BCa Monitor (ABL1, ANXA10, CRH, IGF2, UPK1B)	diagnostic	*n* = 500 (LG = 287, HG = 194)	Cepheid GeneXpert Instrument System was used for sample processing, nucleic acid amplification, and detection of the target sequences. XLSTAT version 2020.2.2 (Addinsoft) was used for data analysis.	BCa recurrence detection		AUC = 0.73, PPV = 21.3%, NPV = 96.5%, Se. = 72.7%, Sp. = 73.7%	retrospective	[41]
Xpert analysis	Diagnostic and prognostic	*n* = 254 (LG = 60, HG = 194)	GeneXpert system (Cepheid, Synnyvale, CA, USA) was used to detect target mRNA sequences (ABL1, ANXA, UPK1B, CRH, IGF2) in urine samples of 254 patients using RT-PCR. Data analysis performed with IBM statistica software v.20.	BCa detection		PPV = 66.4%, NPV = 88.9%, Se. = 85.9%, Sp. = 72.3%	prospective	[42]
CYR61	Prognostic and diagnostic	*n* = 303 (screening set = 30, LG = 12, HG = 18; validation set = 54, LG = 20, HG = 34; FFPE set = 115, LG43, HG = 32; Urine set = 104, LG = 57, HG = 47)	Total RNA was extracted from frozen tissue using TRIzol (Invitrogen, Carlsbad, CA, USA). RNA concentration was determined using a NanoDrop ND-1000 Spectrophotometer (NanoDrop Technologies, Wilmington, DE, USA). RNA’s integrity was determined using a 2100 Bioanalyzer (Agilent Technologies, Santa Clara, CA, USA). qRT-PCR was performed with SYBR PREMIX ex Taq (TaKaRa, Dalian, China) and Mx3005p thermal cycler (Stratagene, La Jolla, CA, USA). Mouse monoclonal antibodies against CYR61 (ab80112; dilution 1:400, Abcam, Cambridge, UK) were used to stain CYR61. Urine samples of 303 patients were tested for CYR61 levels using commercial ELISA test (DY4055, R&D Systems, MN, USA). Data analysis performed with SPSS statistical package, v 17.0 (SPSS Inc., Chicago, IL, USA).	MIBC vs. NMIBC differentiation	BCa progression	AUC = 0.883, Se. = 72.7%, Sp. = 86.0%	retrospective	[43]
Xpert BCa monitor (ABL1, CRH, IGF2, UPK1B, ANXA10)	diagnostic	*n* = 139 (LG = 62, HG = 63)	Urine samples collected from 139 patients were tested with the XBCM. Data analysis peformed with Excel (Microsoft Corp., Redmond, WA, USA).	BCa detection		AUC = 0.79, PPV = 51%, NPV = 92%, Se. = 58%, Sp. = 89%	retrospective	[44]
	Diagnostic and surveillance	*n* = 139 (LG = 139)	Urine samples of 139 patients were tested with Xpert BCa Monitor (Cepheid, Sunnyvale, CA, USA). Data analysis performed with STATA^®^ (IC 16.1; StataCorp LLC, College Station, TX, USA).	BCa detection		No data	prospective	[45]
CxBladder (MDK, HOXA13, CDC2, IGFBP5, CXCR2)	diagnostic	*n* = 28 (LG = 10, HG = 18)	Urine samples collected from 28 patients were sent for urine cytology and CxBladder test. Statistical analysis performed with Microsoft Excel.	BCa detection		PPV = 62%, NPV = 100%, Se. = 100%, Sp. = 75%	prospective	[46]
	diagnostic	*n* = 1411 (Development data set = 863, LG = 43, HG = 46, haematuria = 774; Independent data = 548, LG = 5, HG = 9, haematuria 534)	Quantitative reverse transcription polymerase chain reaction was used on urine of to measure the expression of 5 genotypic biomarkers (MDK, CDK1, IGFBP5, HOXA13, CXCR2). Data analysis was performed with R 3.5.1 software (R Foundation for Statistical Computing, Vienna, Austria).	Stratification of patients at low and high probability of BCa		NPV = 99.4%, Se. = 92.4%, Sp. = 93.8%	prospective	[47]

Abbreviations: AUC—Area under the ROC Curve, *n*—number of patients participating in study, HG—high grade, LG—low grade, BCa—bladder cancer, PCR—polymerase chain reaction, PPV—positive predictive value, qPCR—quantitative polymerase chain reaction, qRT-PCR—Real-Time Quantitative Reverse Transcription PCR, NPV—negative predictive value, Se.—sensitivity, Sp.—specificity, ROBO1—Roundabout Guidance Receptor 1, CRH—Corticotropin Releasing Hormone, IGF2—Insulin Like Growth Factor 2, S100A6—S100 Calcium Binding Protein A6, TRAM1—Translocation Associated Membrane Protein 1, ABL1—ABL Proto-Oncogene 1, ANXA10—Annexin A10, CRH—Corticotropin Releasing Hormone, UPK1B—Uroplakin 1B, CYR61—Cysteine-rich Angiogenic Inducer 61, MDK—Midkine, HOXA13—Homeobox A13, CDC2—Cell Division Cycle 2 (Cyclin Dependent Kinase 1), IGFBP5—Insulin Like Growth Factor Binding Protein 5, CXCR2—C-X-C Motif Chemokine Receptor 2.

**Table 6:** Table presenting data for metabolites biomarkers.

Biomarker	Purpose	Number of Patients	Method	Diagnostic Value	Prognostic Value	Predictive Capacity	Type of Study	Reference
Putative markers	diagnostic	*n* = 124 (BCa = 63, control group = 61)	Urine samples were collected from 124 patients and then derivatizated. GC-MS analysis was performed with Pegasus^®^ 4D GC × GC-TOFMS (LECO, St. Joseph, MI, USA). Data analysis performed with ChromaTOFF^®^ Software (LECO, St. Joseph, MI, USA).	BCa vs. hernia differentiation		AUC = 0.976	retrospective	[52]
Urinary metabolomics	diagnostic	*n* = 44 (BCa = 29, LG = 10, HG = 19; control group = 15)	Metabolomics analysis on urine samples of 44 paitents was conducted with the Q300 Metabolite Assay Kit (Human Metabolomics Institute, Inc., Shenzen, Guangdong, China). Ultra-performance liquid chromatography coupled to tandem mass spectrometry (ACQUITY UPLC-Xevo TQ-S, Waters Corp., Milford, MA, USA) with an electrospray ionization (ESI) source was operated under positive and negative ion modes for the quantitation of metabolites. Data analysis performed with Targeted Metabolome Batch Quantification software (v1.0, Human Metabolomics Institute, Shenzen, Guangdong, China).	BCa detection		AUC = 0.983, Se. = 95.3%, Sp. = 100%	retrospective	[53]

Abbreviations: AUC—Area under the ROC Curve, *n*—number of patients participating in study, HG—high grade, LG—low grade, BCa—bladder cancer, PPV—positive predictive value, NPV—negative predictive value, Se.—sensitivity, Sp.—specificity, GC-MS—gas chromatography-mass spectrometry.

**Table 7:** Table presenting data for others biomarkers.

Biomarker	Purpose	Number of Patients	Method	Diagnostic Value	Prognostic Value	Predictive Capacity	Type of Study	Reference
VPAC	diagnostic	*n* = 103 (group 1 = 65, LG = 30, HG = 35; group 2 = 38, NMIBC)	Urine samples collected from 103 patients were treated with 5-aimnolevulinic acid and then tested for protoporhyrin IX using Nikon ECLIPSE NI fluorescent microscope (Nikon Corporation, Tokyo, Japan). TP4303 solution was used to identify VPAC receptors under fluorescent microscope. All patients underwent cystoscopy/biopsy/TURBT and surgical samples were examined by a pathologist and then compared to the results of conventional cytology, 5-ALA-induced fluorescent cytology, and fluorescent microscopic VPAC receptors examination. Data analysis was performed using Microsoft Excel 2019.	Detection of BCa	n/a	Se. = 89.23%, Sp. = 100%	prospective	[54]
utDNA	Prognostic and diagnostic	*n* = 57 (BCa = 42, MIBC = 32, NMIBC = 10, control group = 15)	Urine and blood sample of 57 patients acquired on the day of RT. cfDNA was isolated from urine and purified by AMPure XP (Beckman Coulter Life Sciences, Indianapolis, IN, USA) and then analyzed by Agilent 2100 Bioanalyzer (Agilent Technologies, Santa Clara, CA, USA). utDNA detection using urine Cancer Personalized Profiling by Deep Sequencing (uCAPP-Seq). Data analysis performed with RStudio v1.1.463 environment (RStudio, Boston, MA, USA) and Prism 8 (GraphPad Software, San Diego, CA, USA).	MRD detection	Predicting FPS and OS	AUC = 0.78, PPV = 88%, NPV = 72%, Se. = 81%, Sp. = 81%	Retrospective	[55]
CP	diagnostic	*n* = 273 (cohort 1 = 97; cohort 2 = 176)	Urine collected from 273 patients was measured for CP levels using commercial ELISA kit (R&D systems, Minneapolis, MN, USA). Data analysis was performed using StatView (v 5.0, Abacus Concepts, Berkeley, CA, USA)		BCa progression	No data	retrospective	[56]
MTC-PCR	prognostic	*n* = 123 (LG = 37, HG = 86)	Urine samples of 123 patients collected after TURBT and before initiation of BCG instillation, and every year after the last BCG instillation, including induction and maintenance for up to 10 yr. DNA was purified from the pellet using a QIAamp DNA kit (Qiagen, Hilden, Germany). MTC-PCR was used to detect the presence of mycobacterial DNA in urine samples. Statistical analysis performed with R software v.3.5.1 (R Foundation for Statistical Computing, Vienna, Austria).		BCa progression and recurrence	**Progression** AUC = 0.875 Se. = 83.3%, Sp. = 91.5% **Recurrence** AUC = 0.868, Se. = 75%, Sp. = 100%		[57]
Multiple Chromatographic Analysis (Fluorescent peak F)	diagnostic	*n* = 47 (BCa = 23, LG = 19, HG = 4; NMHU = 24)	Urine collected from 47 patients was filtered using a 0.22um nylon membrane filter-LLG Syringe Filter PTFE (AZ chrome, Bratislava, Slovak Republic). Urines samples were analyzed using RP-HPLC system Prominence 20A (Shimadzu Co., Kyoto, Japan). Data analysis was performed using Software LC solution (Shimadzu Co., Kyoto, Japan).	BCa vs. NMHU differentiation		AUC = 0.824, PPV = 78%, NPV = 88%, Se. = 90%, Sp. = 74%	retrospective	[59]
miR-34a-5p, miR-205-3p, miR-210-3p	diagnostic	*n* = 147 (prospective: BCa = 15, NMIBC = 12, MIBC = 3, LG = 8, HG = 7; control group = 16; retrospective: BCa = 66, NMIBC = 56, MIBC = 10, LG = 25, HG = 41; control group = 50)	BenchMark XT immunostainer (Ventana Medycal Sysems, Tucson, AZ, USA) was used for immunohistochemical staining of tissue samples. The stains were inspected using an Olympus BX50 and Olympus BX46 mucroscopes (Olympus Europe) by two pathologists. Total RNA was extracted from urine supernatant samples of 116 patients using the Urine microRNA Purification kit (Norgen Biotek, Thorold, Canada). RNA concentration was quantified by Invitrogen Qubit^®^ 4 Fluorometer with Qubit^®^ microRNA Assay Kit (Invitrogen, Milan, Italy). Small RNA transcripts were converted into barcoded cDNA libraries with the NEBNext Multiplex Small RNA Library Prep Set for Illumina (New England Biolabs, Ipswich, MA, USA) and run on Illumina NextSeq 500 platform (Illumina, San Diego, CA, USA). Differential expression analysis was performed with DESeq2 Bioconductor’s package (version 1.22.2). miRNA biomarkers were replicated using miRCURY LNA miRNA PCR Assays (Qiagen, Milan, Italy). Reverse transcription was performed using the miRCURY LNA RT kit (Qiagen, Milan, Italy). Functional enrichment analysis of miRNA target genes was performed using RBiomirGS v0.2.12. Data analysis was performed using Graphpad Prism 8 sofware (Graphpad Software, San Diego, CA, USA) and Quasar-Orange software (Bioinformatics Laboratory of the University of Ljubljana).	BCa detection		AUC = 0.92	Prospective and retrospective	[60]
Urinary VOC analysis	Diagnostic and surveillance	*n* = 305 (BCa = 96, Control group = 209)	Urine samples of 305 patients were collected before cystoscopy. Sulphuric acid solution (Fisher Scientific, Waltham, MA, USA) was added to urine. A PerkinElmer Clarus 500 GC-MS single quadrupole system (PerkinElmer, Waltham, MA, USA) and PAL COMBI-xt autosampler (CTC Analytics, Zwingen, Switzerland) were used to analyse samples. Data analysis performed with Metaboanalyst.	BCa detection		**Eight-Voc diagnostic biomarker** AUC = 0.77, Se. = 71%, Sp. = 72% **Six-VOC surveillace biomarker** AUC = 0.80, Se. = 71%, Sp. = 80%	retrospective	[61]

Abbreviations: AUC—Area under the ROC Curve, n—number of patients participating in study, HG—high grade, LG—low grade, BCa—bladder cancer, PCR—polymerase chain reaction, PPV—positive predictive value, NPV—negative predictive value, Se.—sensitivity, Sp.—specificity, cfDNA—cell-free DNA, utDNA—urine tumour DNA RP-HPLC—reverse-phase high-performance liquid chromatography, ELISA—enzyme-linked immunosorbent assay, uCAPP-Seq—urine Cancer Personalized Profiling by Deep Sequencing, VPAC—Combined vasoactive intestinal peptide and pituitary adenylate cyclase-activating peptide family of cell surface receptors, utDNA—urine tumour DNA, CP—Ceruloplasmin, MTC-PCR—Mycobacterium tuberculosis complex polymerase chain reaction, miR—microRNA, VOC—Volatile Organic Compounds.

## Data Availability

Not applicable.

## Abbreviations

ANG	Angiogenin
AUC	Area under the curve
AS	Active surveillance
BC	Bladder cancer
BCG	Bacille Calmette–Guérin
BE	Bladder EpiCheck
BIAS	Bladder Cancer Italian Active Surveillance
BTA	Bladder tumour antigen
BTG2	B cell translocation gene 2
cfDNA	Cell-free DNA
CIS	Carcinoma in situ
CK	Cytokeratin
CS	Cystoscopy surveillance
CSS	Cancer-specific survival
CYFRA 21.1	Cytokeratin fragment 21.1
DDX17	DEAD-Box Helicase 17
DFS	Disease-free survival
GC-MS	gas chromatography-mass spectrometry
GC-TOFMS	gas chromatography coupled to a time-of-flight mass spectrometer
EAU	European Association of Urology
EMA	European Medicines Agency
EV	Extracellular Vesicles
FFS	Failure free-survival
HG	High grade
HPLC	high-performance liquid chromatography
IHC	Immunohistochemistry staining
iTRAQ	Isobaric tags for relative and absolute quantitation
LC-MS	liquid chromatography-mass spectrometry
LG	Low grade
lncRNA	Long non-coding RNAs
MDR	Minimal residual disease
MIBC	Muscle-invasive bladder cancer
NMIBC	Non-muscle invasive bladder cancer
NMP22	Nuclear matrix protein 22
NMR	nuclear magnetic resonance
NPV	Negative predictive value
OPLS-DA	Orthogonal Projections to Latent Structures Discriminant Analysis
OS	Overall survival
pCR	pathologic complete response
PD-L1	Programmed death-ligand 1
PFS	Progression-free survival
PPV	Positive predictive value
qMSP	quantitative Methylation Specific PCR
SERS	surface-enhanced Raman spectroscopy
TERC	Telomerase RNA component
TURBT	Transurethral resection of bladder tumour
UBC	Urothelial bladder cancer
UBT	Urinary biomarker test
UC	Urothelial carcinoma
UPLC-MS	Ultra-performance liquid chromatography coupled to mass spectrometry
VOC	volatile organic compounds
WLC	White-light cystoscopy
